# The Role of Chain Molecular Weight and Hofmeister Series Ions in Thermal Aggregation of Poly(2-Isopropyl-2-Oxazoline) Grafted Nanoparticles

**DOI:** 10.3390/polym10040451

**Published:** 2018-04-17

**Authors:** Martina Schroffenegger, Ronald Zirbs, Steffen Kurzhals, Erik Reimhult

**Affiliations:** Institute for Biologically Inspired Materials, Department of Nanobiotechnology, University of Natural Resources and Life Sciences Vienna, Muthgasse 11, 1190 Vienna, Austria; martina.schroffenegger@boku.ac.at (M.S.); ronald.zirbs@boku.ac.at (R.Z.); steffen.kurzhals@boku.ac.at or steffen.kurzhals@ait.ac.at (S.K.)

**Keywords:** poly(2-isopropyl-2-oxazoline), lower critical solution temperature (LCST), superparamagnetic iron oxide nanoparticles (SPION), thermoresponsive polymer, reversible nanoparticle aggregation, Hofmeister series, spherical polymer brush, dynamic scanning calorimetry (DSC), dynamic light scattering (DLS)

## Abstract

Thermoresponsive nanoparticles are promising smart materials for many applications. However, a rational design for applications requires a deeper understanding and experimental verification of the various parameters that influence the thermoresponsiveness of the spherical polymer brushes that define most of such nanomaterials. Therefore, we investigate superparamagnetic iron oxide nanoparticles (SPION) grafted with poly(2-isopropyl-2-oxazoline) (6–33 kg mol^−1^) by temperature-cycled dynamic light scattering and differential scanning calorimetry. The grafting of dense spherical polymer brushes leads to lower aggregation temperatures and transition enthalpies when compared with the free polymer. The transition enthalpy and temperature depend on the polymer shell size and structure. The addition of kosmotropic salts decreases the aggregation temperature following the Hofmeister series.

## 1. Introduction

Nanoparticles grafted with thermoresponsive polymers have received much attention in recent years because of the multitude of applications for which they are of interest. Thermoresponsive core-shell nanoparticles are multifunctional materials, combining the nano-specific properties of the core with the thermoresponsive solubility of the shell. For example, Kakwere et al. report iron oxide cubes grafted with poly(*N*-isopropylacrylamide) (PNIPAM), which can be used as drug delivery vehicles and for hyperthermia treatment [1]. Such smart materials can be used not only for drug delivery [2,3,4], but also for magnetic separation [5], water desalination [6], or catalysis [7]. These applications require a detailed understanding of how thermoresponsive (reversible) aggregation of colloidally stable polymer-grafted nanoparticles can be controlled under relevant conditions. The impact of the grafting of thermoresponsive polymers on nanoparticles is still poorly investigated experimentally. This is mainly because of a lack of suitable model systems for which nanoparticle structure can be rationally designed and investigated, and which are colloidally stable under physiological conditions.

While nanoparticles grafted with poly(ethylene glycol) (PEG) dominate research in the practical use of non-responsive biomedical nanoparticles, PNIPAM-based systems account for the vast majority of publications in the field of thermoresponsive nanoparticles, despite the fact that PNIPAM has a thermal solubility transition in water that is below body temperature. However, for biomedical applications, poly(2-oxazolines) have emerged as a true alternative to PEG because of their non-toxic and biocompatible nature [8] in the face of mounting antibody production to PEG [9] and the stability of poly(2-oxazolines) to hydrolysis [10]. In addition, poly(2-oxazolines) can be designed with a critical solution temperature (CST), above which they lose hydration. Through the monomer composition of poly(2-oxazolines), the CST can be designed to be close to or above body temperature [11,12,13]; these combined properties make them potentially much more versatile for biotechnological and biomedical applications than PNIPAM or PEG.

Oxazolines are polymerized via cationic ring-opening polymerization (CROP), which allows for the synthesis of polymers with defined molecular weights (MW), composition, and end-group chemistry, as well as narrow molecular weight distribution [14]. Poly(2-isopropyl-2-oxazoline) (PiPOx)—a structural isomer to PNIPAM—exhibits a lower critical solution temperature (LCST) in water at a few degrees below body temperature [15,16,17]. During this thermally triggered solubility transition, the polymer undergoes a phase change from a soluble to a phase-separated state, driven mainly by entropy. The LCST describes the lowest temperature in the compositional phase diagram at which this transition occurs. The CST of a hybrid polymer system, such as nanoparticles grafted with polymers, therefore, can significantly deviate from the LCST, as the local concentration and imposed conformation of the polymer in such systems reflect other parts of the phase diagram [18].

Several factors are known to shift the CST of PiPOx in addition to the concentration of the polymer [19,20], including the mixing of salt with the aqueous polymer solution [21], the variation of end-groups [22], and varying the polymer MW [20,23]. Short PiPOx chains (1.7 kg mol^−1^) display higher transition temperatures (72.5 °C) than longer chains (5.7 kg mol^−1^, 48.1 °C) [23]. The enthalpy of the transition increases significantly with the MW [23]. The concentration of free polymer has been shown to have smaller influence on the CST of PiPOx in water than that of the MW. Park et al. studied the cloud point of PiPOx with a MW of 9.7 kg mol^−1^. Changing the concentration of the polymer from 0.1 to 1.0 wt % resulted in a decrease of the cloud point from 39.8 to 37.3 °C. Furthermore, the impact of the concentration on the CST decreases with increasing MW [19]. The CST can be lowered by introducing a hydrophobic end-group to a polymer, whereas it increases with a hydrophilic end-group. For example, by changing a methyl end-group to a nonyl end-group, the CST of PiPOx was lowered by 19 °C [22]. It also worth noting that the CST of PiPOx can be manipulated by copolymerization. The CST can be shifted downward by having more apolar moieties present [24]. By incorporating shorter chains, such as ethyl or methyl moieties, the CST will increase to higher values when compared with PiPOx [12].

The addition of salts to the aqueous polymer solution plays a crucial role in shifting the CST of thermoresponsive polymers. Many—in particular, medical, biotechnological, and environmental—applications require the function of responsive particles at both high ionic strength and in the presence of a large variety of different salts. The logic for the influence of salts is simple; salts are known to affect the internal hydrogen bonding of water, and thus strongly influence the solubility transition of polymers such as PiPOx, which is driven by the balance of water hydrogen bonding and entropy. Therefore, kosmotropes decrease the CST as the ionic strength is increased, while chaotropes will increase the CST. Previous studies on free PiPOx have found that kosmotropic anions decrease the transition temperature. With increasing salt concentration, a linear decrease in CST was observed. On the other hand, chaotropic anions increase the CST nonlinearly when the ion concentration is increased [21,25]. For poly(2-ethyl-2-oxazoline) (PEtOx)—which is structurally similar to PiPOx, but has higher LCST—mainly three mechanisms were found that cause a change in transition temperature. In the presence of chaotropic anions (NO_3_^−^, I^−^, ClO^−^, SCN^−^), a direct binding of the anions to the polymer is observed, leading to an increase in polymer solubility, tantamount to a shift of the CST to higher values. Divalent, kosmotropic anions (CO_3_^2−^, SO_4_^2−^, S_2_O_3_^2−^) effectively desolvate the polymer chains and lower the CST. Monovalent, kosmotropic anions (H_2_PO_4_^−^, F^−^, Cl^−^, Br^−^) cause desolvation and increase the surface tension of the solution. Therefore, the addition of monovalent, kosmotropic anions causes an increase in the free energy of the alkyl/water interface, leading to lower solubility and lower CST of the polymer [25]. Much less attention has been paid to the influence of cations on poly(2-oxazoline) CST, but they have been claimed to have less impact on CST than anions [21,25].

Moreover, the polymer architecture has a significant influence on the CST. Star-shaped PiPOx display a lower aggregation temperature when compared with its linear analogs. Eight-arm, star-shaped PiPOx with a MW of 19.6 kg mol^−1^ aggregate at a concentration of 2 g L^−1^ at 37 °C, while the linear analog of one arm (MW 2.5 kg mol^−1^) aggregates at 47 °C [26]. It should, however, be noted that a more relevant comparison would require linear polymers of the same MW as the star-shaped polymer in order to investigate the influence of the branched topology. A strong effect of the architecture on the CST is also observed for comb copolymers [27,28,29]. As expected, the CST can be modulated both by the MW of the side chain and by the length of the main chain [27,28,29].

Core-shell nanoparticles with a spherical PiPOx shell are topologically and morphologically similar to star-shaped polymer architectures [30]; thus a similar effect on the CST of the grafting of spherical brushes on nanoparticles as that of adding arms to a star-shaped polymer is expected. In a previous report, the thermal aggregation and deaggregation of poly(2-oxazoline)-grafted iron oxide nanoparticles were investigated with respect to shell composition and exposure to the physiological buffer and medium [31]. Three different poly(2-oxazoline) shells—PiPOx, PEtOx, and a random copolymer PiPOx-*co*-PEtOx in 87:13 ratio—were investigated. In all cases, the CST of the polymers shifted to lower values upon grafting. By adding the cell culture medium, which contains a high concentration of the kosmotrope NaCl, the critical solution temperature of the grafted PiPOx (16.5 kg mol^−1^) brush dramatically shifted lower to 32.5 °C, compared to 38 °C in pure water. Analogue particles, with 14 kg mol^−1^ grafted PEtOx at a concentration of 1 g L^−1^, showed a decrease in CST from 74 °C in pure water to 47 °C in cell culture medium [31]. However, an investigation into the influence of chain MW of PiPOx and ionic strength (type and concentration of ions) on the CST of the spherical brushes was not performed.

Given the potential applications of poly(2-oxazoline)-grafted nanoparticles, it is crucial to explore the impact of surface grafting, brush morphology, and the presence of ions on their thermal responsiveness. Therefore, we investigated monodisperse, superparamagnetic iron oxide nanoparticles regarding the influence of grafting, chain MW, and type of anions and cations from the Hofmeister series on the thermoresponsive properties of the PiPOx shell and on the thermally-induced aggregation of the nanoparticles. Differential scanning calorimetry (DSC) and dynamic light scattering (DLS) were used to elucidate the influence of the unique internal structure, provided by grafting of the linear PiPOx into a nanoscale spherical brush, on the thermal colloidal properties.

## 2. Materials and Methods 

### 2.1. Materials

All of the chemicals were purchased from Merck KGaA (Darmstadt, Germany) and used without further purification, unless noted otherwise. Methyl-*p*-tosylate was purified by distillation. COMU (≥99%, Carl Roth, Karlsruhe, Germany): (1-cyano-2-ethoxy-2-oxoethylidenaminooxy)dimethylamino-morpholino-carbenium hexafluorophosphate. Dialysis tubes with a molecular weight cut-off (MWCO) of 3.5 kDa (regenerated cellulose) and 1000 kDa (cellulose ester, Spectra/Por^®^ Float-A-Lyzer) were purchased from Carl Roth. The synthesis of oleic acid-coated SPION was described previously [32,33]. Shortly, iron oxide nanoparticles were synthesized by thermal decomposition of iron(0)pentacarbonyl in the presence of oleic acid as a surfactant.

**Synthesis of 2-isopropyl-2-oxazoline:** The monomer was synthesized using a modified Witte−Seeliger cyclocondensation [34,35]. In brief, 1 equivalent (eq.) (0.5 mol, 45 mL) of isobutyronitrile was reacted with 1.2 eq. (0.6 mol, 36 mL) of ethanolamine in the presence of 0.02 eq. (0.01 mol, 2.2 g) zinc acetate dihydrate as catalyst at 130 °C for 24 h. The product was dissolved in dichloromethane and purified by extraction with water until the pH of the aqueous phase was neutral. It was dried over calcium hydride and distilled in an inert atmosphere.

**Polymerization of 2-isopropyl-2-oxazoline:** General synthesis of the polymer: In a flame-dried vial, a 25 vol % solution of 2-isopropyl-2-oxazoline (2 mL) in anhydrous dimethylacetamide (DMA) (6 mL) was mixed with an appropriate amount ([M]:[I] = 51–288) (60–10 µL) of methyl-*p*-tosylate and stirred at 100 °C for up to 24 h. The polymerization was quenched with water (10 eq.). The polymer was isolated by precipitation with diethyl ether (Et_2_O) and hexane (ratio 1:1). The MW was determined by gel permeation chromatography against polystyrene standards.

**Carboxy-terminated polymer (PiPOx-COOH):** As a representative reaction, PiPOx-21 will be presented. The functionalization of the polymer was prepared according to Kurzhals et al. [31]. In dry chloroform, 10.3 eq. (1.3 mmol, 128.9 mg) of succinic anhydride and 3.4 eq. (0.43 mmol, 52.9 mg) of DMAP were added to a solution of PiPOx (1 eq., 17.7 mmol, 2 g) and refluxed for 24 h. The polymer was isolated by precipitation with Et_2_O and hexane.

**Nitrochatecol-terminated polymer (PiPOx-NDA):** The reaction of PiPOx-21 is presented as a representative synthesis. Carboxy terminated PiPOx (1 eq., 0.10 mmol, 2.09 g) was activated by 3.8 eq. (0.38 mmol, 162.1 mg) COMU and 12 eq. (1.2 mmol, 200 µL) *N,N*-diisopropylethylamine (DIPEA) in dry dimethyl formamide (DMF) for 10 min in an inert atmosphere. A solution of 6-nitrodopamine hydrogen sulfate (4.2 eq., 0.42 mmol, 126 mg) in DMF was slowly added and stirred for 48 h. The polymer was precipitated into Et_2_O/*n*-hexane (*v*/*v*: 1/1). The excess of 6-nitrodopamine was removed by dialysis (MWCO: 3.5 kDa). Degree of functionalization 70%. Overall yield: 1.3 g, 65%.

^1^H-NMR for PiPOx-NDA δH (300 MHz; CD_3_OD) 7.53 (1H, s, Ar–H), 6.71 (1H, s, Ar–H), 4.27 (2H, CH_2_OCO–), 3.53 (4nH, –N–CH_2_CH_2_– PIPOX), 3.03–2.77 (1nH, CH(CH_3_)_2_, PIPOX), 1.10 (6nH, CH(CH_3_)_2_, PIPOX).

**Ligand exchange on SPION (“grafting to” of PiPOx):** The reaction of PiPOx-21 is shown as a representative sample. In 0.5 mL toluene, 401 mg wet oleic acid-coated SPION (inorganic fraction 5%) was dissolved. In 12 mL DMF, 1.292 g nitrocatechol-terminated PiPOx-21 was dissolved. Both solutions were mixed and reacted under ultrasonication for 48 h. The particles were precipitated with Et_2_O/hexane (ratio 1:1) and dialyzed against water (MWCO: 1000 kDa) for 4 days. Yield: 121 mg.

### 2.2. Methods

^1^H-NMR spectra of polymers were measured on a BRUKER AV III 300 MHz spectrometer (Brucker Austria GmbH, Vienna, Austria). Chemical shifts were recorded in ppm and referenced to a residual protonated solvent (CDCl_3_: 7.26 ppm (^1^H) and CD_3_OD: 3.31 ppm (^1^H)). Polymer MWs were measured by GPC on a Malvern Viscotek GPCmax system. The setup comprised of three MZ Gel SDPlus columns (a precolumn followed by two columns with separation ranges of 10–2000 kDa and 1–40 kDa, respectively). A Knauer Smartline RI Detector 2300 (Dr. Ing. Herbert Knauer GmbH, Berlin, Germany) was used to detect the difference in refractive indices. DMF with 0.05 M LiBr was applied as the eluent. Samples of 50 µL with a concentration of 3 g L^−1^ were injected and measured at 60 °C at a flow rate of 0.5 mL min^−1^. OminSEC 5.12 was used for analysis. Polystyrene standards of 1.5–651 kg mol^−1^ were used for external calibration. IR spectra of lyophilized samples were recorded on a Bruker Vertex 70 FTIR spectrometer (Bruker Austria GmbH, Vienna, Austria) at a resolution of 4 cm^−1^, averaging 32 scans. Transmission electron micrographs were recorded on a FEI Tecnai G2 (FEI Europe B.V., Austria), with 160 kV acceleration voltage on carbon-coated grids. Nanoparticle size distributions were calculated with the freeware Pebbles [36], based on the analysis of >2000 nanoparticles. Thermal gravimetric analysis of the core-shell nanoparticles was performed on a Mettler Toledo TGA/DSC (Mettler Toledo GmbH, Vienna, Austria), with 80 mL min^−1^ synthetic air as reactive gas, 20 mL min^−1^ nitrogen as protective gas and a heating rate of 10 K min^−1^ from 25 to 650 °C. Using TGA results, the grafting density (σ) was calculated using the following formula:

$$\sigma =\frac{{\left(\text{\% }w/w\right)}_{shell}\;\rho_{iron\;oxide\;}V_{core}\;N_{A}}{{\left(\text{\% }w/w\right)}_{core}\;M_{polymer\;}A_{core}},$$

where σ is the grafting density; ${\left(\%\;w/w\right)}_{shell}$ is percentage of mass loss in TGA for the organic fraction corresponding to the polymer grafted to the iron oxide core; $N_{A}$ is the Avogadro constant; $\rho_{iron\;oxide}$ is the density of iron oxide; $V_{core}$ is the volume; $A_{core}$ is the area of the iron oxide core, calculated from the diameter of the cores measured by TEM; $M_{polymer}$ is the MW of polymer; and ${\left(\%\;w/w\right)}_{core}$ is the residual mass percentage of the inorganic fraction in TGA. DLS measurements ($D_{H}$, CST, and temperature cycling experiments) were conducted in Milli-Q water on a Malvern Zetasizer Nano-ZS. Mean values and standard deviation of the count rate and the number-weighted diameter were calculated from three measurements for each temperature step. Temperature-cycled experiments were performed in the temperature range from 25 to 50 °C with a step-size of 1 °C. After changing the temperature, the sample was equilibrated for 2 min and then measured three times. Each reported value was an average of 11 runs. The CST was determined by the onset of the increasing curve of count rate versus temperature. Microdifferential scanning calorimetry measurements on nanoparticle dispersions in Milli-Q water were performed using a Malvern MicroCal PEAQ-DSC Automated system. Data processing was done using the MicroCal PEAQ-DSC software version 1.22. The CST was in analogy to the determination via DLS determined by the onset of a peak. Statistical analysis was carried out with DataLab (Version 3.530). The transition enthalpies per monomer unit were calculated from DSC heating curves, using data from TGA and GPC. TGA gives the polymer weight fraction of the PiPOx-grafted SPION, which can be used to calculate the molar amount of PiPOx in the dispersed SPION sample used for DSC. The number average molecular weight of PiPOx was measured by GPC. Division by the monomer MW gives the degree of polymerization. The monomer transition enthalpy is then calculated using the following formula:

$$\Delta H_{monomer}\left[{\mathrm{kJ}\;\mathrm{mol}}^{-1}\right]=\frac{Peak\;area\left[\mathrm{kJ}\right]}{n_{PiPOx}\left[\mathrm{mol}\right]\;DP},$$

where $\Delta H_{monomer}$ corresponds to the average transition enthalpy per monomer unit, Peak area corresponds to the integral of the DSC thermogram under the measured curve, $n_{PiPOx}$ corresponds to the molarity of the polymer, and DP corresponds to the degree of polymerization or number of monomer units in the polymer.

## 3. Results and Discussion

### 3.1. Synthesis of PiPOx-NDA Grafted Superparamagnetic Iron Oxide Nanoparticles (SPION)

**Scheme 1:**
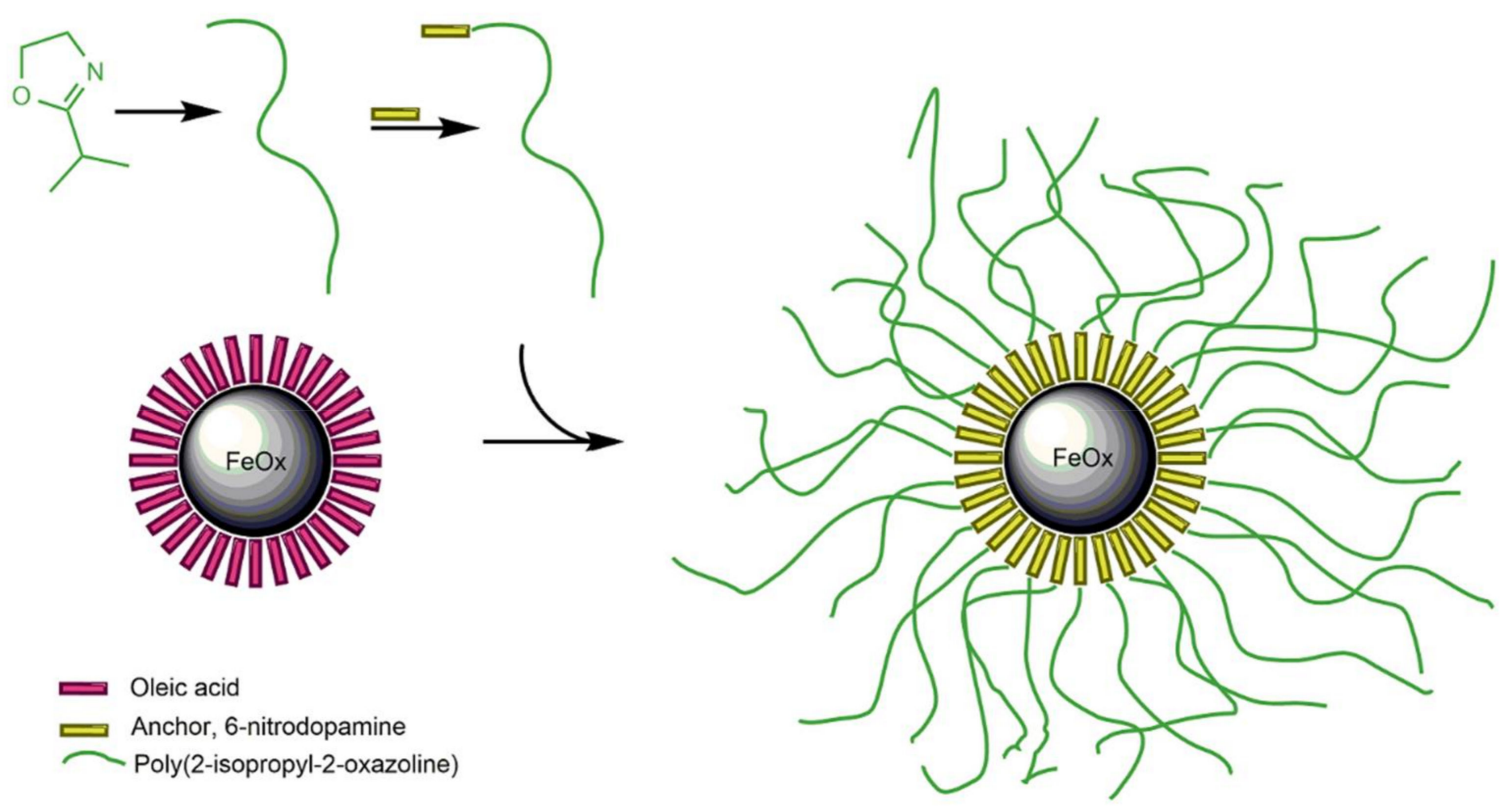
Overview of the preparation of thermoresponsive particles.

Four different core-shell nanoparticles with identical core diameters (9.1 ± 0.3 nm), but different PiPOx dispersant MWs ranging from 6 to 33 kg mol^−1^, were prepared to investigate the influence of the PiPOx MW on the thermoresponsiveness. The polymers were synthesized by living cationic ring-opening polymerization, using methyl-*p*-tosylate as an initiator. The MWs were determined by GPC, against polystyrene standards. As an anchor to graft the PiPOx chains irreversibly to the SPION surface, we selected 6-nitrodopamine (NDA) [37]. The binding of NDA exhibits a covalent character to metal oxide surfaces with especially high affinity to Fe^III^ [38,39,40]. NDA-modified polymers were reacted with oleic acid-coated SPION in a “grafting to” process, described in Section 2 and Scheme 1. With this method, SPION with a spherical, thermoresponsive brush with grafting densities of 0.8–1.0 PiPOx chains per nm^2^ can be generated.

All of the samples dispersed spontaneously in water over a broad concentration range up to 10 g L^−1^ and showed excellent colloidal stability due to the irreversibly bound and dense spherical brush. The particles did not aggregate in the solution after one month at room temperature. The characteristics of the free polymers and polymer-grafted SPION are summarized in Table 1. As expected for constant grafting density (σ) the hydrodynamic diameter ($D_{H}$) of the dispersed nanoparticles increases with the MW of the polymer dispersant. Thermal gravimetric analysis (TGA) data, infrared (IR) spectra, and transmission electron microscopy (TEM) images of the SPION grafted with PiPOx are provided in the Supporting Information (Figures S1–S3).

### 3.2. Temperature-Induced Aggregation of Free Linear PiPOx Coils

The thermal responsiveness of free linear polymer coils was studied at a polymer concentration of 1 g L^−1^ by DLS and DSC. The two techniques differ in that the CST is determined by DLS as the colloidal aggregation leading to increased scattering of light and change of the average colloid size. This is equivalent to a very sensitive measurement of the cloud point temperature that can also be applied at concentrations and for samples that show no visible increase in turbidity. DSC measures the CST as the temperature at which the specific heat capacity required to break water hydrogen bonds to the polymer upon the desolvation peaks. An overview of the results is presented in Table 2. As expected, there was a clear trend of decreasing CST with increasing PiPOx MW. The smallest polymer (6 kg mol^−1^) displayed the highest transition temperature of all of the investigated samples, with a CST of 46 °C estimated by the colloidal transition observed by DLS at this temperature. This was in agreement with values reported by Diab et al. of 48.1 °C for PiPOx with a MW of 5.7 kg mol^−1^ [23], measured by turbidity measurements at a polymer concentration of 1 g L^−1^. DSC yielded similar CSTs when compared to DLS, except for PiPOx-6 (the smallest MW), for which the CST was significantly higher when measured by DSC. The minor ~1 °C higher CST measured by DSC when compared with DLS for the other samples could be explained by the different heating rates that were applied for the respective techniques. For the DSC measurements, a continuous heating rate of 1 K min^−1^ was used. This was faster than for DLS, for which the temperature was changed in 1 K steps at ~7 min intervals, as was required to accumulate sufficient scattering statistics for each data point.

**Figure 1:**
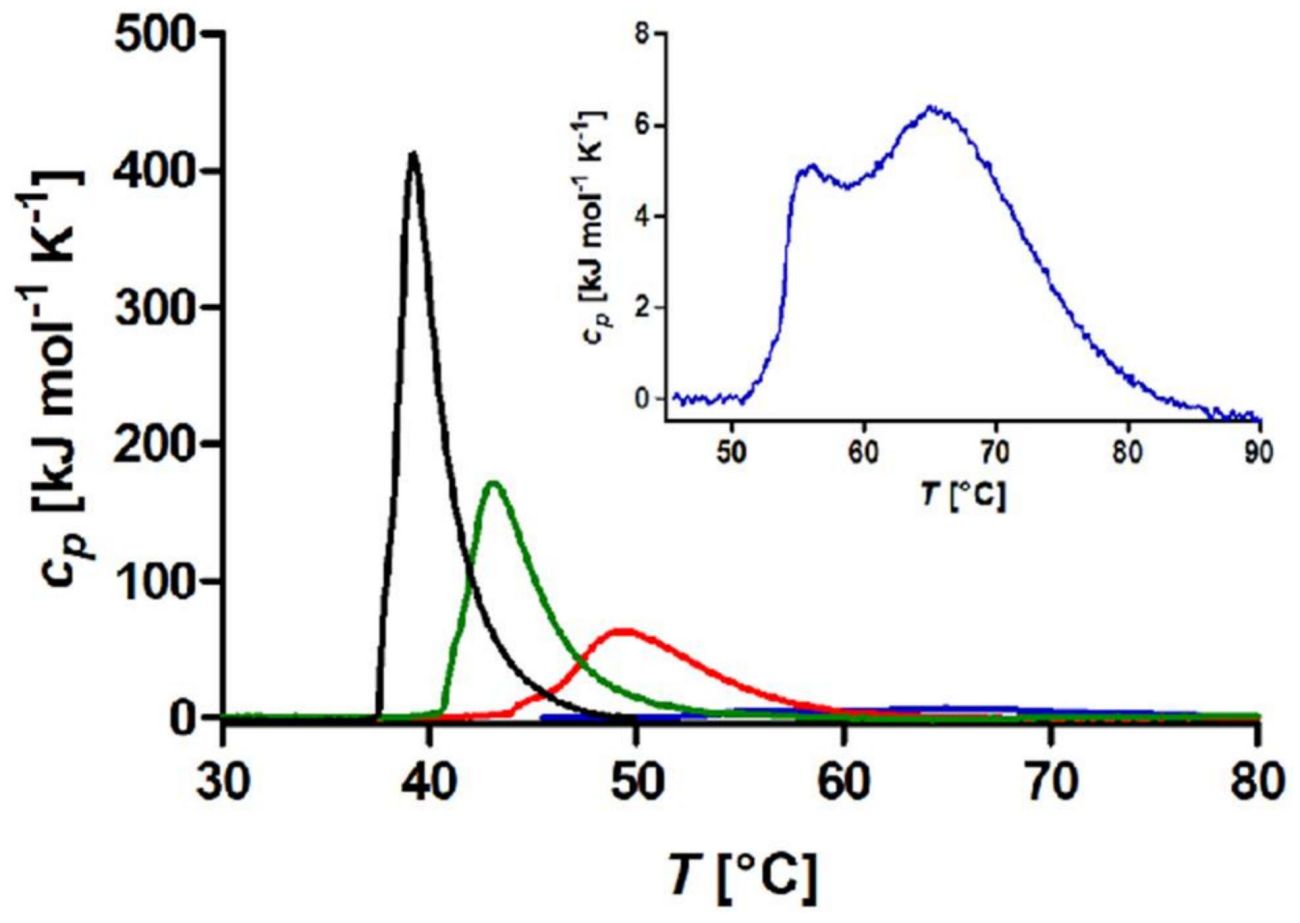
Dynamic scanning calorimetry (DSC) curves for solutions of free polymers in Milli-Q at a concentration of 1 g L^−1^ and a heating rate of 1 K min^−1^. The specific heat capacity ($c_{p}$) was normalized by the polymer concentration. All curves were background subtracted and fitted with a spline baseline. Blue: Poly(2-isopropyl-2-oxazoline) (PiPOx-6), red: PiPOx-14, green: PiPOx-21, black: PiPOx-33. Inset: DSC curve of PiPOx-6 plotted with smaller T and $c_{p}$ ranges.

A comparison of the DSC curves for the free polymers is provided in Figure 1. It clearly indicates decreasing CSTs with increasing MW, as well as a narrowing of the temperature range over which the main transition took place. The narrowing is an indication of higher cooperativity of the breaking of the polymer–water H-bonds for higher MW. The transition enthalpies obtained by the integration of the endothermic peaks and normalized per monomer unit range from 1.9 kJ mol^−1^ for the lowest MW to approximately 4.2 kJ mol^−1^ for all of the other MWs (Table 2). The transition enthalpies per monomer of the higher MW PiPOx (≥14 kg mol^−1^) were all on the order of a weak hydrogen bond, while the lowest MW PiPOx (6 kg mol^−1^) demonstrated a transition enthalpy per monomer of only half of a weak hydrogen bond. PiPOx monomers form two or more hydrogen bonds to water [41], of which only one has been suggested to break during the desolvation transition [23]. On the other hand, theoretical calculations of the thermal solubility transition of PiPOx have shown that the number of hydrogen bonds decreases by 12.4% from a hydrated state at 20 °C to a dehydrated state at 60 °C [42]. Our results were roughly in this range, with the lowest MW of 6 kg mol^−1^ corresponding closely to the theoretical prediction, while the higher MWs (14–33 kg mol^−1^) corresponded closely to other experimental findings [23,42]. However, it is notable that the 6 kg mol^−1^ free PiPOx had a very broad transition peak (see inset of Figure 1).

A closer look at the DSC curves reveals a sharp increase in the heat capacity ($c_{p}$) at the onset of the peak; after reaching the maximum, a weaker decrease of $c_{p}$ was visible, which ended in a long tailing. This asymmetry fitted with previous studies on the thermal solubility transitions of PiPOx. These studies focused especially on the conformational changes of the polymer upon heating. Below the CST, each monomer unit can bind two or more water molecules through hydrogen bonding and the polymer takes a conformation in which *trans* and *gauche* bonds coexist [41]. With increasing temperature, the hydrogen bonds are partially cleaved and replaced by weaker polymer–water–polymer bridging [43], inducing a liquid–liquid phase separation. This cleaving of the hydrogen bonds to water could induce the sharp increase in heat capacity observed in the DSC. Continued heating resulted in the breaking of more hydrogen bonds and the formation of free carbonyl (C=O) groups, followed by the breaking of methyl carbonyl (CH_3_∙∙∙O=C) bonds [41,43,44]. The further cleavage of weaker intermolecular hydrogen bonds at higher temperatures also contributed to the transition enthalpy. The many parts of the transition, together with cooperativity effects and the non-uniform distribution of monomers through the polymer coil that are pronounced for higher MW polymers, can explain the broad and asymmetric peaks in heat capacity.

### 3.3. Temperature-Induced Aggregation of Dispersed SPION Grafted with PiPOx at Constant Mass Concentration

**Figure 2:**
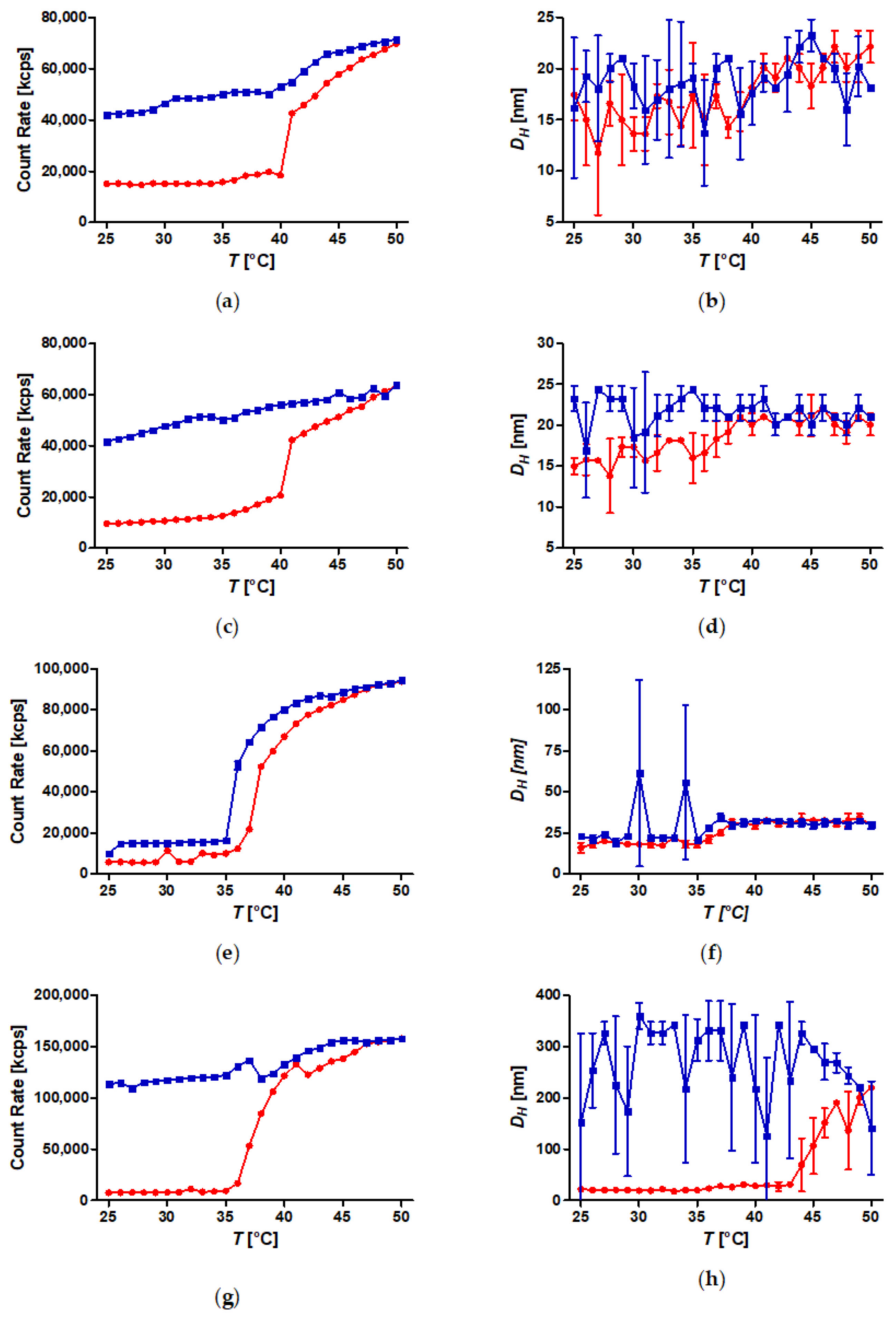
Temperature-cycled dynamic light scattering (DLS) for superparamagnetic iron oxide nanoparticles (SPION) dispersions in Milli-Q at a concentration of 1 g L^−1^, FeOx-6: (**a**,**b**); FeOx-14: (**c**,**d**); FeOx-21: (**e**,**f**); and FeOx-33: (**g**,**h**). **Left**: count rate vs. temperature, **right**: hydrodynamic diameter ($D_{H}$) vs. temperature, heating curve (red circles), cooling curve (blue squares). Mean values and standard deviations of the count rate and number-weighted diameter were calculated from three measurements for each temperature step.

Next, the influence of the grafting of PiPOx as spherical brushes on monodisperse superparamagnetic iron oxide nanoparticles with a core diameter of 9.1 ± 0.3 nm on temperature-induced aggregation was studied using DLS. Figure 2 shows SPION grafted with PiPOx of different molecular weights corresponding to the free polymer coils described in the Section 3.2, monitored at 1 g L^−1^ dispersion in Milli-Q water by DLS. The colloidal stability was studied in the temperature range of 25–50 °C with a step-size of 1 K for both heating and cooling. After changing the temperature, the sample was equilibrated for 2 min and then measured three times. Each reported value was an average of 11 runs. The results are reported in Figure 2. All of the samples displayed a sharp increase in the count rate at a temperature corresponding to the critical solution temperature. The DLS count rate was more sensitive for the detection of the transition temperature than for the change in average sample hydrodynamic diameter ($D_{H}$) because of its strong $D^{6}$ dependence on aggregate size and additional increase due to higher scattering contrast as a denser, collapsed polymer shell is formed.

Of all samples, only FeOx-21 exhibited spontaneous reversible aggregation within the experimental time frame, as measured by both count rate and size (Figure 2e,f). For FeOx-6, FeOx-14, and FeOx-33, the thermal transition of the shell and aggregation upon heating was not fully reversible in the time frame of the measurement. However, the samples spontaneously returned to their initial values after hours at room temperature. In Table 3, the values of $D_{H}\;$ below and above the CST are reported. It can be clearly seen that the hydrodynamic size increased for all SPION samples upon heating. Moreover, SPION with higher MW dispersants exhibited larger cluster size above the CST. A Mann–Whitney *U* test was carried out to test if the differences in $D_{H}$ of the samples below and above the transition temperature were statistically relevant. The test yielded that the change in average particle/cluster diameter was statistically relevant with a level of significance of 95%. This suggests that the thermally-induced collapse of the shell was followed by the formation of small aggregates. Sample FeOx-33 showed a second transition at 43 °C (Figure 2h), which was more pronounced in the hydrodynamic size than in the count rate. This could be interpreted as the formation of large aggregates that even seemed to increase in size upon cooling. That the increase in scattering was less pronounced than the increase in hydrodynamic size determined by the Brownian motion implies that the clustered particles were still well separated by the collapsed shells and predominantly scattered individually.

The sizes of the large clusters after the second transition were not considered for the calculation of $D_{H,\;T>\mathrm{CST}\;}$ in Table 3. The clusters formed upon heating for FeOx-6 and FeOx-14 were small, but did not immediately deaggregate upon cooling (Figure 2a–d). Deaggregation of the clustered SPION requires several hours at room temperature. For sample FeOx-33 (Figure 2g,h), the same behavior of slow deaggregation was observed. It is worth nothing that FeOx-33 formed larger aggregates; the average cluster size was one order of magnitude larger than for FeOx-6 and FeOx-14.

The easily identifiable onset temperature for the main transition in the count rate curve was defined as the CST from the colloidal aggregation measurements. For all of the polymer samples, the CST determined by DLS decreased upon grafting, with the largest difference observed for FeOx-6, for which the CST decreased by 6 °C (Table 2 and Table 3). Furthermore, the CST decreased with increasing MW of grafted PiPOx. The trend of decreasing CST with increasing MW also for grafted polymer chains was in agreement with a previous study on PNIPAM grafted to iron oxide nanoparticles [45]. A brush with a MW of 20 kg mol^−1^ was necessary for immediate spontaneous reversible aggregation/deaggregation of PNIPAM-grafted nanoparticles, which was very similar to our observation for PiPOx.

### 3.4. Temperature-Induced Aggregation of Dispersed SPION Grafted with PiPOx at Constant Molarity

All of our samples had very similar grafting densities, but as previously shown, experimentally and theoretically, the local concentration of polymer radially out from the core varies [30]. The theory for spherical polymer brushes tells us that the segment density can be near constant in the inner parts of the shell at high grafting density, but decreases first polynomially and then exponentially farther out for sufficiently high MW [30]. Therefore, varying the chain MW means varying the average polymer concentration within the polymer shell, as well as the ratio of different polymer conformations within the shell. Varying the MW of the chains also means that at constant mass concentration or molarity, the concentration or volume fraction of polymer varies between the samples. Designing an experiment that compared the influence of only the MW on grafted spherical brushes to nanoparticles without including concentration effects is thus non-trivial. Using DLS, we measured the CST transition through colloidal aggregation, and colloidal aggregation is known to depend significantly on particle concentration. Keeping a constant mass concentration of nanoparticles as the MW was varied, as done in Figure 2, also meant that the molarity of nanoparticles was varied, which would influence the colloidal aggregation kinetics and equilibrium. To limit this possible artefact, the CST should be measured at constant particle molarity. The bulk concentration has been shown to have much less influence than the polymer MW on the CST for free PiPOx coils in this concentration range [19]. The effect of the local polymer crowding on the particles and how chain MW affects this was thus expected to influence colloidal aggregation more at constant particle molarity than the resulting effective difference in global polymer concentration between the samples of different MW. Therefore, we measured temperature-cycled DLS at a constant molarity of 5 × 10^13^ particles per mL. This concentration was chosen so that all sample concentrations decreased compared to 1 g L^−1^. An overview of the results can be found in Table 4 (total count rate and $D_{H}$ as a function of T are shown in Figure S4). The CSTs have shifted to higher values but follow the same trend as at the variable (higher) particle concentrations. A difference in the hydrodynamic diameter for the sample FeOx-6 below and above the CST was difficult to observe by eye (Figure S4b), but the Mann–Whitney *U* test showed that the size difference was statistically relevant with 95 % significance.

Sample FeOx-14 exhibited unexpected behavior. It formed significantly larger aggregates when compared with results obtained at higher concentrations (Figure 2d and Figure S4d). This could hint at low colloidal stability of the suspended particles. Although similar, FeOx-14 had a slightly lower grafting density than the other samples (cf. Table 1). A lower grafting density can influence the aggregation, in particular by allowing cores to have stronger attractive van-der-Waals interactions with each other. At low grafting density, the steric distance between the cores introduced by the collapsed polymer shell was smaller when compared with that at high grafting densities, and it also might have been more inhomogeneous. The aggregation might even have become irreversible if the grafting density decreased below a critical value [46].

**Figure 3:**
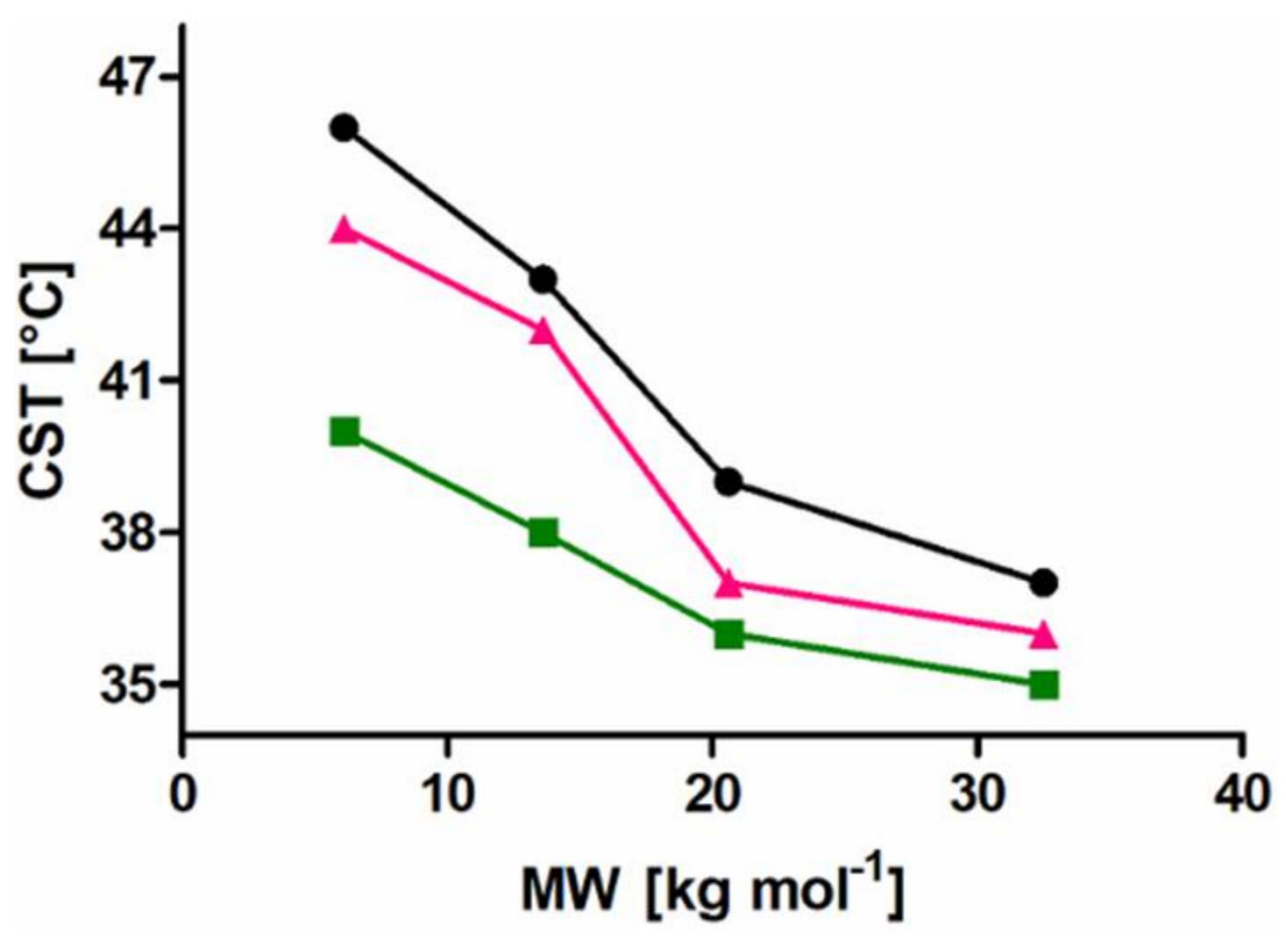
Summary of the critical solution temperatures as function of PiPOx molecular weight (MW) for free polymers and PiPOx-grafted SPION measured in Milli-Q water by DLS as the temperature at which aggregation starts (the onset of the transition in the count rate curve). Black circles: Free PiPOx (1 g L^−1^), pink triangles: PiPOx-grafted SPION (5 × 10^13^ particles per mL), green squares: PiPOx-grafted SPION (1 g L^−1^).

In Figure 3, the CSTs for free polymers and SPION are plotted against the dispersant MW. In agreement with previous reports in the literature [31,47,48,49], it was shown that the CST shifted to lower values when the polymer was grafted to nanoparticles, and additionally, that this was true regardless of MW. Figure 3 also indicates that the aggregation temperature was decreased for polymer-grafted nanoparticles by increasing the particle concentration, since the samples at constant molarity all had lower concentration than the constant mass samples; while this dependence seemed high for SPION grafted with low molecular weight PiPOx, the dependence seemed to become weaker as the grafted PiPOx molecular weight was increased.

### 3.5. Temperature-Induced Aggregation of FeOx-21 at Different Concentrations

**Figure 4:**
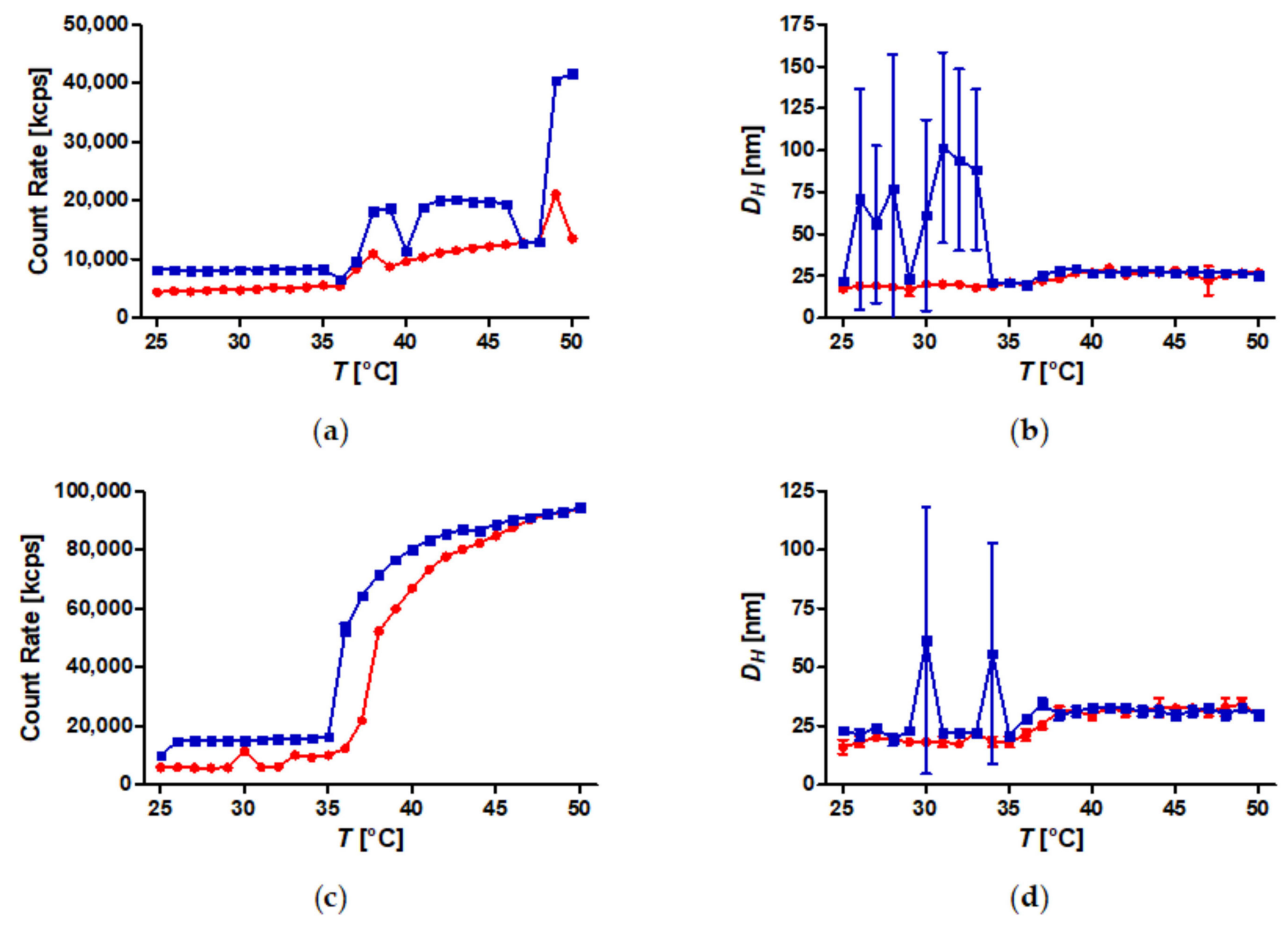

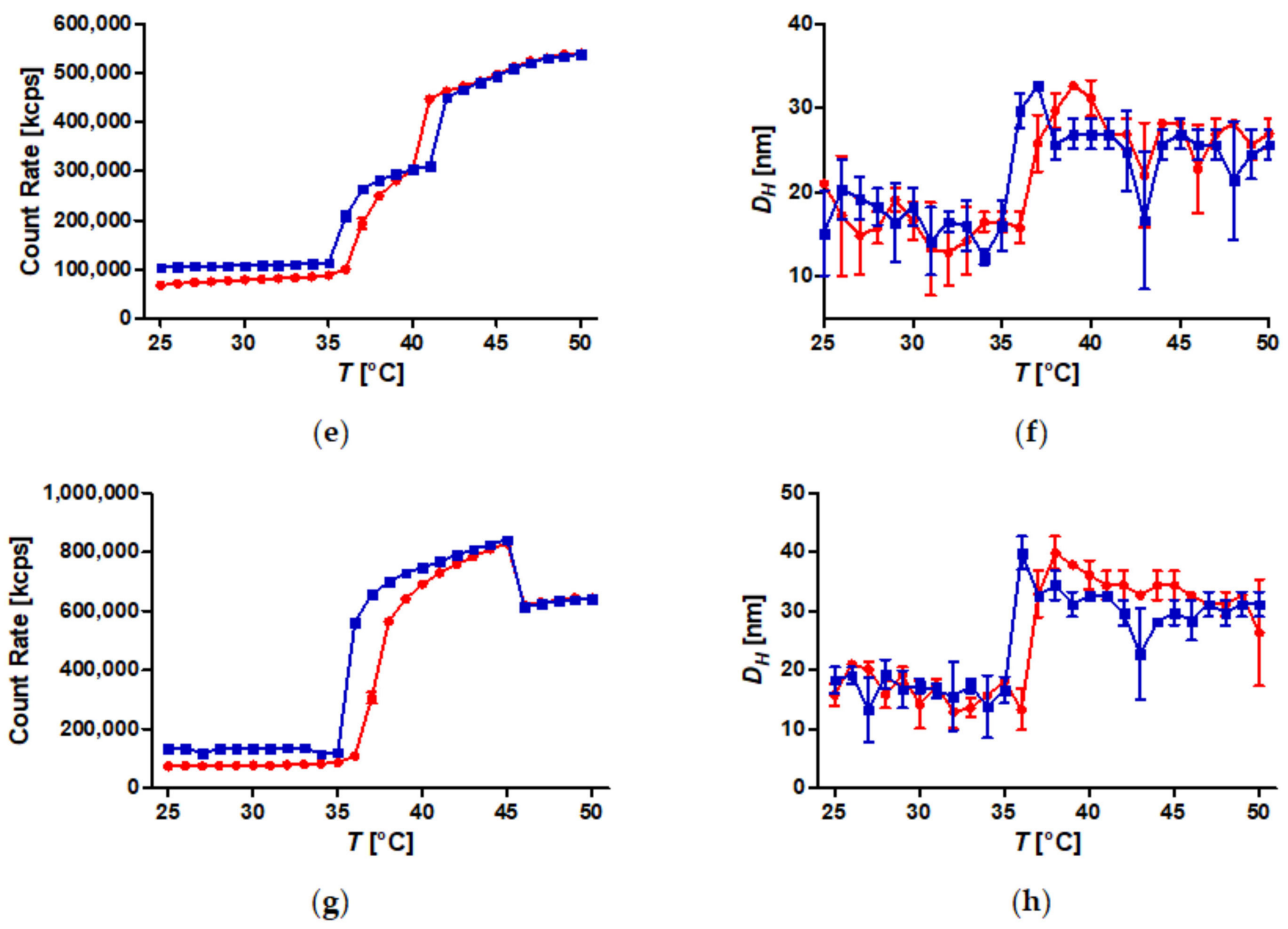
Temperature-cycled DLS for dispersions of FeOx-21 in Milli-Q at different concentrations. **Left**: count rate vs. temperature, **right**: hydrodynamic diameter ($D_{H}$) vs. temperature. (**a**,**b**): 0.54 g/L; (**c**,**d**): 1 g L^−1^; (**e**,**f**): 5 g L^−1^; (**g**,**h**): 10 g L^−1^. Heating curve (red circles), cooling curve (blue squares). Mean values and standard deviations of the count rate and number-weighted diameter were calculated from three measurements for each temperature step.

Given the results reported in Figure 3 that indicate a strong dependence of CST on nanoparticle concentration, we expanded the phase diagram of nanoparticles grafted with a polymer to additional different concentrations of one of the samples. FeOx-21 was chosen because it was the only sample showing reversible behavior during the time frame of a DLS cycle. Figure 4 shows reversible aggregation of small clusters at all concentrations for FeOx-21. At the lowest concentration of 0.54 g L^−1^, the DLS size measurements were sensitive to minute amounts of larger aggregates, causing large standard deviations in the size estimates after cooling. The cluster hydrodynamic diameter above CST, $D_{H,T>\mathrm{CST}\;}$, was independent of the sample concentration and corresponded only to ~30 nm concurrent with no visible turbidity (i.e., the clusters contained only a few nanoparticles). Varying the particle concentration from 0.54 to 10 g L^−1^ for FeOx-21 also did not significantly change the CST. Only the lowest concentration showed a marginally higher CST of 37 °C compared to 36 °C at all other concentrations.

These results were consistent, but they seemed to be an outlier compared to the data for the other MW samples reported in Figure 3. They also contrasted the strong concentration dependence reported for free PiPOx of lower MW (4.3 kg mol^−1^), for which the CST changed from 57 °C to 49 °C by increasing the concentration from 0.5 to 5 g L^−1^ [23]. Further, Zhao et al. studied PiPOx of different MWs over a wide concentration range, from 0.2 to 85 wt %, and reported CSTs as low as 26.1 °C at 19.8 wt % for PiPOx with a MW of 13 kg mol^−1^ [17]. A smaller impact of the concentration on the CST was observed for star-shaped polymers. An eight-arm star-shaped PiPOx (MW: 19.6 kg mol^−1^) showed a CST of 37.1 °C at a concentration of 2 g L^−1^, which was decreased to 30 °C by increasing the concentration to 12 g L^−1^. The CST only decreased by another three degrees to 27 °C when the concentration was increased to 32 g L^−1^ [26].

The decrease in the CST of star polymers when compared with their linear analogs was explained by having a high local polymer density. The ability of star polymers to form H-bonds was similar to that for the polymer at high concentrations because of the steric constraints in star-shaped polymers for segments close to the core [50]; this decreased the CST and made the CST less sensitive to further increases in global polymer concentration. The same argument is valid for spherical brushes grafted on core-shell nanoparticles, since they have an analogous structure [30]. The constant value of the CST with respect to concentration is thus an indication of the very high local concentration of polymer in the spherical brush grafted to the nanoparticle surface and its reduction of hydrogen bonding within the polymer brush; it could even mean that we were measuring a CST close to the LCST of PiPOx-21, despite a global polymer fraction below the one at which the LCST was expected to be observed. The apparent concentration dependence of CST of at least the lower MW PiPOx-grafted nanoparticles is observed in Figure 3, which, in contrast to the in-depth study of the concentration dependence for FeOx-21, could be because of either a strong MW dependence or that these samples have residual free polymer. We note that the CSTs measured for the core-shell nanoparticles were far from those reported for the free polymer (cf. Table 1, Table 2, Table 3 and Table 4) and that polymers with lower MW generally showed a stronger concentration dependence. Additionally, as described in the Section 3.6, the DSC measurements did not imply any free polymer in the lower MW samples, while FeOx-21 is likely to have contained some excess free polymer. This made a MW dependence of the effect of the global polymer fraction on the CST the most plausible explanation for our results, since Figure 3 indicates that the concentration dependence of the CST was also weak for FeOx-33.

### 3.6. Comparison of Enthalpic Transition Measured by DSC and Colloidal Aggregation of PiPOx-Grafted SPION

**Figure 5:**
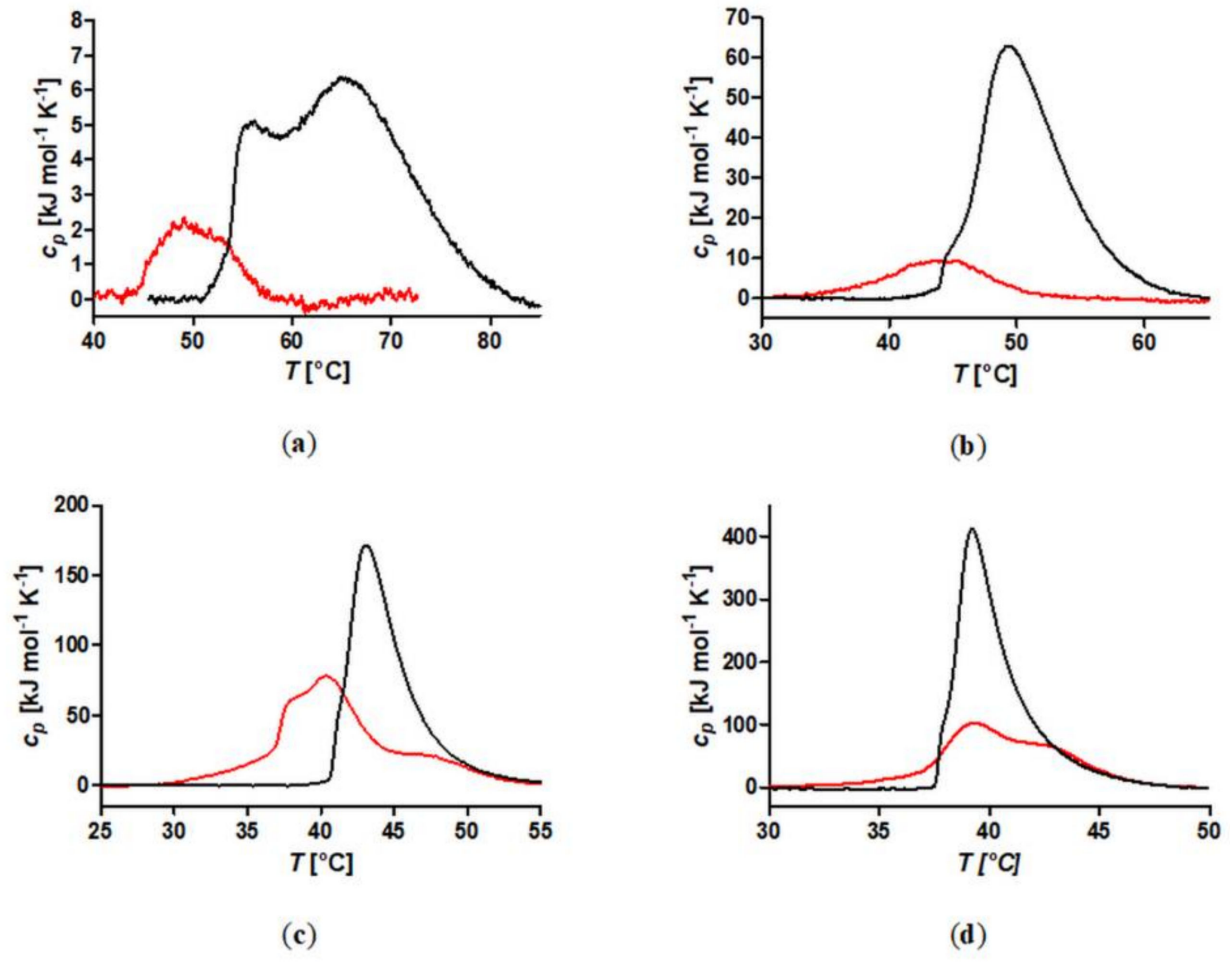
Overlay of DSC curves for free PiPOx and SPION grafted with PiPOx. Concentrations of free polymer solutions and SPION dispersions in Milli-Q were 1 g L^−1^ and a heating rate of 1 K min^−1^ was used. (**a**) Black: PiPOx-6, red: FeOx-6; (**b**) black: PiPOx-14, red: FeOx-14; (**c**) black: PiPOx-21, red: FeOx-21; (**d**) black: PiPOx-33, red: FeOx-33.

The DLS measurements showed that the grafting of PiPOx to a spherical brush on nanoparticles changed the CST and affected the aggregation behavior. Given that the polymer density and brush morphology varied within a spherical polymer brush, DSC could provide significant additional insights; it could detect variations in the thermal transition within the brush due to these differences, which might not all manifest themselves in colloidal aggregation. Figure 5 shows DSC curves of free PiPOx coils in water for each molecular weight compared with measurements of SPION with the respective PiPOx grafted as a spherical brush shell. For each MW, the transition enthalpy per monomer unit of the SPION sample was smaller than for the corresponding free linear polymer coil. This effect was already observed for SPION grafted with PNIPAM, for which grafted chains displayed smaller transition enthalpies when compared with their free analogues [45].

We also observed that the data for the SPION samples appeared to comprise multiple transitions, observed as convoluted transition peaks giving rise to broadened peak shapes and shoulders. The complete thermal transitions of the nanoparticle dispersions were thus fitted using a fixed number of Gaussians ($T_{peak\;max}$, $c_{p,max}$, and the variance used as free parameters). While the total transition peak shape could be reproduced with a low residual error for FeOx-6 and FeOx-14, the two SPION grafted with higher molecular weight PiPOx had to be fitted with three peaks. FeOx-21 additionally displayed a minor peak at a significantly higher temperature than the main transitions (Figure 5c), which required the fitting of an additional fourth Gaussian peak. We assigned this peak with a peak maximum temperature of 47 °C to a low amount of residual free polymer, since it was well separated and corresponded closely to the transition measured for the free polymer, but shifted to a slightly higher T tentatively because of the lower concentration. The other samples showed no similar indication of free polymer judging from the separation of the individual peaks for free PiPOx and those constituting PiPOx-grafted SPION. In agreement with the results by DLS, DSC also demonstrated the shift of the CST to lower values upon grafting (Figure 5a–c).

The enthalpy, calculated by the integration of the endothermic peak and normalized per monomer unit gave the added heat required for the phase transition. The transition enthalpy for the grafted brushes (Table 5) increased with the MW of the PiPOx polymer dispersant from 0.4 kJ mol^−1^ for PiPOx-6 to 2.5 kJ mol^−1^ for FeOx-21, but it was smaller than in the free polymer state in all cases (cf. Table 2 and Table 5). This agreed with the results obtained for PiPOx homopolymers and block copolymers grafted to SPION [31,51].

**Figure 6:**
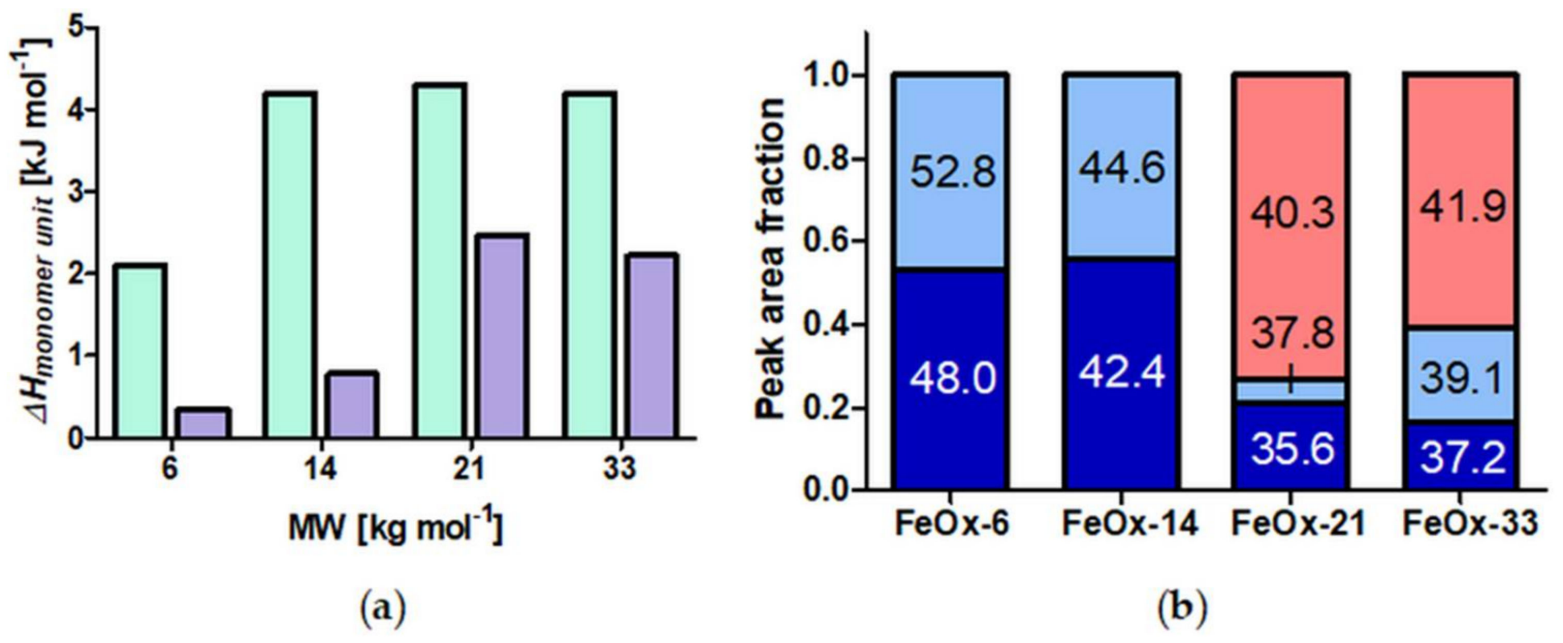
(**a**) Enthalpy per monomer unit for free and grafted PiPOx as a function of MW. The ΔH was derived from the integration of the corresponding DSC curve. $\Delta H_{monomer}$ is the normalized value per monomer unit. Green: free polymer, purple: core-shell nanoparticle; (**b**) Fractions of the enthalpy of the transition attributed to different parts of the shell by fitting multiple peaks to the specific heat capacity as a function of the temperature for SPION grafted with PiPOx. The DSC curves for the main transition were fitted with 2–4 Gaussian curves. The contribution of each fitted Gaussian was normalized. The value in each bar was the temperature at the maximum of each fitted transition. The contributions correspond to the first (dark blue), second (light blue), and third (red) fitted peaks, where the first and second peaks were part of a broad first transition and the third peak fits a distinct second transition in the DSC curves. The fourth peak corresponding to residual free polymer in the dispersion of FeOx-21 was not included.

In relative terms, the enthalpy was reduced from the free polymer state by ~80% for FeOx-6 and FeOx-14, but only by ~45% for FeOx-21 and FeOx-33 (Figure 6a). In a previous publication, it could be demonstrated that brush segments closer to the core experienced a stronger reduction in their thermoresponsiveness, including CST and enthalpy, when compared with outer shell segments [51]. With increasing PiPOx MW, the fraction of outer shell segments was increasing and thus a greater fraction of shell segments was in a less sterically constrained state. This provided a clear rationale for the fact that the transition enthalpy per monomer moved closer to that of a free coil as the MW of grafted PiPOx was increased, as observed in Figure 6a. For FeOx-33, a slight drop in the transition enthalpy when compared with FeOx-21 was observed. This unexpected difference additionally supported the interpretation of the DSC curve for the FeOx-21 dispersion as that residual free polymer was present, since free polymer had a higher transition enthalpy per monomer than grafted polymer chains.

Cooling curves for free PiPOx and PiPOx-grafted SPION were also recorded with a 1 K min^−1^ cooling rate. For the free polymers, the peak areas extracted from the heating and respective cooling curves were of a similar magnitude, showing the full reversibility of the solubility transition. The results are summarized in Table 6. The transition enthalpies derived from the cooling curves for samples PiPOx-14 and PiPOx-21 even exceeded the transition enthalpies of the preceding heating run, which indicates that the polymer might not have been in an equilibrated solvation state at the beginning of the heating run. During the heating, the chain segments underwent conformational changes (erasing thermal history), making more chain segments accessible for resolvation in the cooling run. The peak maximum of the free polymer samples shifted to lower temperatures in the cooling curve. This hysteresis ($\Delta T_{max})$, however, decreased with molecular weight and was negligible for PiPOx-33 (Table 6).

A similar trend for the dependence of the main transition temperature on molecular weight and temperature sweep direction was observed for PiPOx grafted to SPION. The two higher molecular weight samples, FeOx-21 and FeOx-33, did not exhibit any hysteresis, while the low molecular weight PiPOx samples, FeOx-6 and FeOx-14, showed 4 and 8 °C lower transition temperatures, respectively, during the cooling sweep. The significant undercooling for FeOx-14 indicated slow resolvation. This correlated with this sample having the lowest grafting density and it could thus potentially have been explained by increased particle/particle attraction in the desolvated state, which counteracted the resolvation. The transition enthalpy upon cooling was lower when compared with the transition enthalpy measured in the heating run for all SPION dispersions. It is possible that only the shell segments on the exterior of the small particle clusters resolvated in the time frame of the DSC cooling run. (NB. The different shell transitions also could be observed in the cooling sweep at similar ratio as in the heating sweep). The polymer inside the nanoparticle clusters required a longer time to resolvate because of restricted water diffusion. FeOx-21 showed the highest rehydration and the fastest reversibility in the time frame of the T-cycled DLS measurements. This, again, was in agreement with the interpretation that some free polymer was present in the FeOx-21 sample, which facilitated complete rehydration by creating more loosely packed clusters. It was interesting to observe that for all SPION dispersions, a small, but significant, fraction of the brush was rehydrated, as evidenced by the reforming of PiPOx–water hydrogen bonds within minutes. However, the clustering of the particles and increased core–core interaction appeared to prevent the natural fast rehydration of PiPOx in water upon cooling, as evidenced by the redispersion times measured by DLS.

As described above, we fitted the thermal transitions of the PiPOx-grafted SPION with several peaks, where the transitions for FeOx-6 and FeOx-14 were well fitted by two peaks and the DSC curves for FeOx-21 and FeOx-33 required three peaks to fit the main transition. The fits are presented in the Supporting Information in Figure S6. While the DSC thermograms of free coils had a tail that required the fitting of multiple Gaussian peaks for a good fit (cf. Figure 1 and Figure S5), it was clear that this was the result of a gradient of polymer coil properties in one single transition. The two Gaussian peaks that fitted to all SPION dispersions at lower temperatures were convoluted in a single broad peak, which was not well described by a single Gaussian, just like for the free polymer. However, the third fitted peak, observed only for FeOx-21 and FeOx-33, was distinct and clearly observable as a separate maximum in the curve. The broadening also observed for free polymers could originate from breaking different types of H-bonds during the transition that differ in strength and thus in transition temperature [43]. However, the transition temperatures for different types of H-bonds were reported to differ only by 1 °C, while the peak maxima for FeOx-21 and FeOx-33 were separated by ~5 °C. Thus, multiple convoluted peaks identified by DSC were likely to derive from differently hydrated shell segments.

Therefore, we attributed the first two fitted peaks to be part of the transition of the inner part of the PiPOx brush shell, where the segment density was high and slowly decreasing with distance from the core [30,52]. The onset of the dehydration transition, measured by DSC, was at 31 °C for all core-shell SPION, except FeOx-6 (see Table 5). This further suggested attributing the convoluted two first fitted peaks to the inner part of the shell, given the low transition temperature and that the most conserved conformation of a spherical brush should be found close to the core as the molecular weight is varied. We attributed the third fitted peak to an extension of the polymer shell into an outer part with significantly lower segment density and thus a transition temperature closer to that of free polymer coils. While recognizing that any transition between regimes had to be gradual, this nonetheless motivated the fitting of multiple peaks and attributing them to different polymer conformations in the spherical brush shell. The data strongly supported the existence of two rather distinct regions within the spherical polymer brush that had different conformations and hydration strengths. Tentatively, the broad inner shell transition corresponded to a dense brush-like regime, while the outer part corresponded to a mushroom-like regime that was more similar to a free coil.

Similar results were reported by Shan et al. who observed two different transitions in DSC measurements for gold nanoparticles grafted with PNIPAM [53]. The first transition was assigned to the inner shell segment close to the core, which was a densely packed brush and thus in a low hydration state below the CST. The second transition was assigned to an outer segment exhibiting coil-like conformation with corresponding higher hydration [53]. This two-step transition was observed for a shell with a PNIPAM MW of around 5 kg mol^−1^. We only observed the same phenomenon for SPION grafted with PiPOx of MWs >14 kg mol^−1^. This could have been because of differences in other parameters that affected the brush morphology and thereby the ratio between strongly restricted monomers in the brush to monomers in more coil-like segments, such as grafting density, hydrophobicity of the end-group, and brush curvature (nanoparticle core size).

Figure 6b displays the relative enthalpy of the transitions of the different parts of the shells as estimated from integrating the fitted peaks attributed to each transition. The red bars correspond to the outer “mushroom” shell and the blue bars correspond to the inner “brush” shell. Roughly half of the enthalpy of the total thermal transition for the SPION grafted with higher molecular weight PiPOx appeared to be attributed to the outer part of the shell. Since we observed a reduction in the average transition enthalpy per monomer for grafted versus free coil polymer, it is likely that the fraction of the polymer belonging to the outer part of the shell was lower than this estimate. How large the outer part of the shell was had significance for understanding the colloidal transitions in comparison to the observed thermal hydration transitions. An outer part of the shell with a high transition temperature could have still stabilized the particles and prevented strong aggregation even if the inner shell dehydrated and collapsed at a low temperature. This also appeared to be observed for the FeOx-33 sample. Figure 2h and Figure S8 display two transitions in the colloidal aggregation state that corresponded well with the transition temperatures measured by DSC for the same sample. Strong aggregation into very large aggregates was only observed after the second transition at 42–43 °C, which corresponded well to the second transition observed for the outer part of the shell by DSC.

### 3.7. Temperature-Induced Aggregation of Dispersed SPION Grafted with PiPOx at Different Salt Concentrations

**Figure 7:**
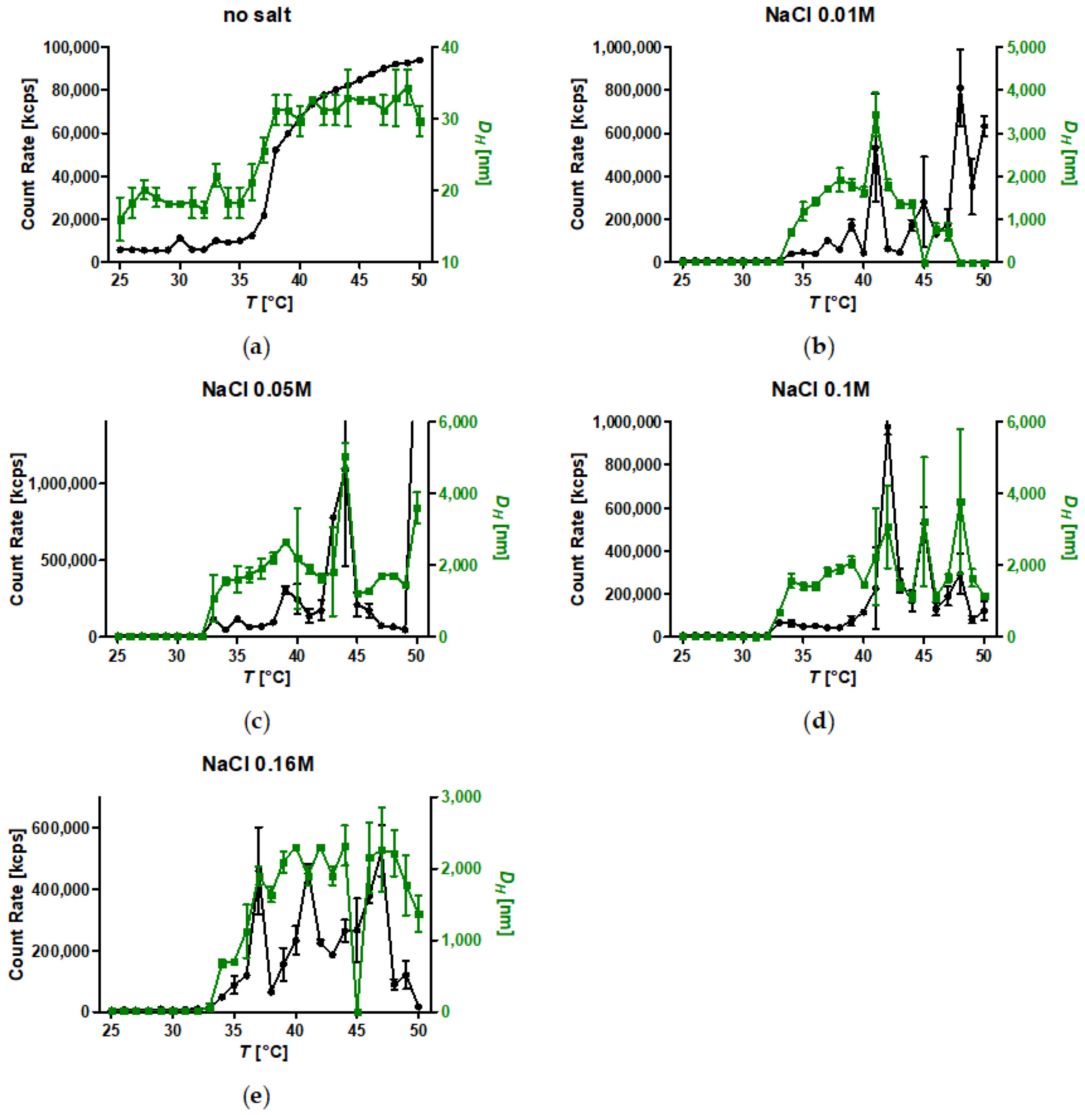
DLS-heating curves for dispersions of SPION FeOx-21 (1 g L^−1^) in NaCl solutions of different molarity. (**a**) no salt; (**b**) 0.01 M NaCl; (**c**) 0.05 M NaCl; (**d**) 0.1 M NaCl; (**e**) 0.16 M NaCl. Black circles: count rate, green squares: hydrodynamic diameter ($D_{H}$). Mean values and standard deviations of the count rate and number-weighted diameter were calculated from three measurements for each temperature step.

Studying thermal polymer transitions and colloidal aggregation in pure water represents an ideal case that does not closely resemble the environment in biological media, which, for example, have high concentrations of different types of salts. Heating curves of SPION dispersions (1 g L^−1^ FeOx-21) containing different amounts (0.01, 0.05, 0.1, 0.16 mol L^−1^) of salt were measured to investigate the effect of ionic strength on the CST. Figure 7 presents the DLS data for dispersions of SPION in the various NaCl solutions as a function of temperature.

The addition of NaCl decreased the CST when compared with pure water. At the lowest NaCl concentration (0.01 mol L^−1^), the CST decreased by 3 °C. By increasing the concentration of NaCl to 0.16 mol L^−1^, a decrease of 4 °C when compared with Milli-Q water was observed. A significant difference between the measurements in water and NaCl solution could be observed in the aggregation of the investigated SPION too. In the presence of NaCl (≥0.05 mol L^−1^) aggregates in the micron range (2–4 µm) were formed upon heating (Figure 7b–d); these large aggregates precipitated during the measurement (Figure 7), while reversible aggregation and deaggregation was observed for dispersions in Milli-Q (Figure 2 and Figure S7). The large aggregates were likely a result of the increased hydrophobic energy penalty of the desolvated polymer brushes in the presence of kosmotropes, causing a further decrease of the exposed polymer brush/water interfacial area by clustering. These large aggregates could only be redispersed by shaking. Precipitates were formed that could not be resuspended at all if the samples containing NaCl were kept above the CST for longer than 10 h. These precipitates were possibly stable because of crystallization of the PiPOx [41,44].

**Figure 8:**
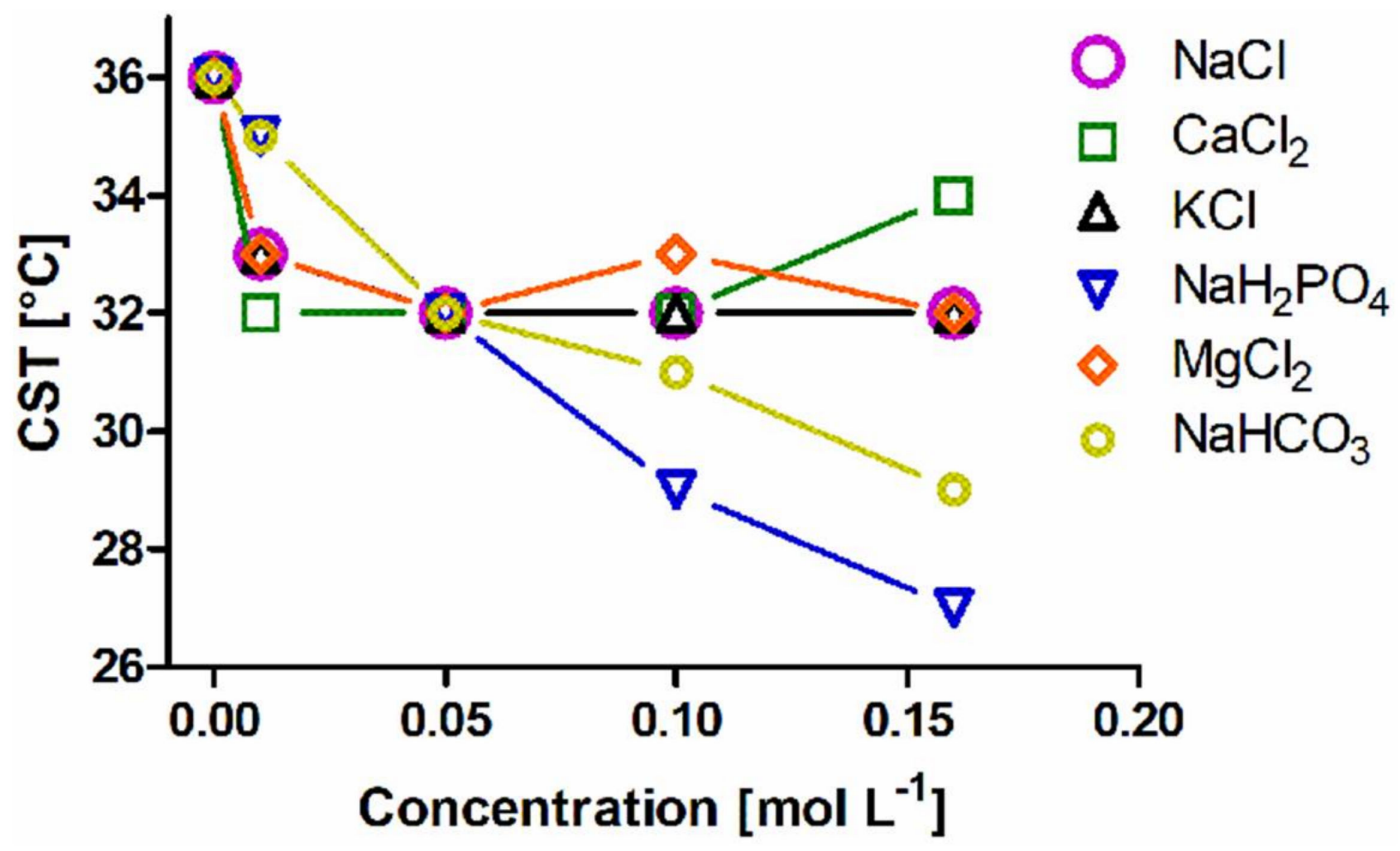
Critical solution temperature of dispersions of FeOx-21 (1 g L^−1^) in salt solutions as a function of ionic strength. The data were extracted from the corresponding DLS count rate vs. temperature curves.

Five salts in addition to NaCl were chosen with ions from the Hofmeister series: KCl, MgCl_2_, CaCl_2_, NaH_2_PO_4_, and NaHCO_3_; these were also investigated regarding their impact on the thermal solubility transition of FeOx-21. The respective DLS measurements are provided as Figures S9–S13 and the observed dependence of the CST on the respective salt and salt concentration is presented in Figure 8.

All of the investigated salts contained kosmotropic anions. Anions such as HCO_3_^−^ and H_2_PO_4_^2−^ exhibited a stronger influence on the aggregation of the core-shell nanoparticle than Cl^−^, which was in agreement with their position in the Hofmeister series: H_2_PO_4_^−^ > HCO_3_^−^ > Cl^−^. Similar findings were reported for free PEtOx [25]. By having a 0.1 M concentration of H_2_PO_4_^−^ present in the solution, the CST was lowered by 8 °C. We could observe a similar decay of 7 °C for 0.1 M H_2_PO_4_^−^. In contrast, varying the counter cations in our study did not influence the CST significantly, as seen by the almost identical curves obtained for the chloride salts NaCl, KCl, MgCl_2_, and CaCl_2_. CaCl_2_ showed a slightly larger decrease of the CST at low concentrations when compared with the other chloride salts and a slight increase in CST for the highest salt concentration; this indicates a slight effect of its place in the Hofmeister series as a chaotropic cation. When comparing the monovalent and divalent cations, it should be noted that the samples have different molarities of anions; however, in the measured concentration range, the higher molarity of anions clearly plays no significant role, since the measured transitions were around 32 °C over almost the entire concentration range. For free PiPOx, no significant difference between Li^+^ and Na^+^ was found [21]. Overall, it can be concluded that the cations of the tested kosmotropic salts played a minor role when compared with the anions regarding shifting the CST of PiPOx densely grafted on nanoparticles. Furthermore, although the reduction of the CST due to kosmotropes was significant, it was smaller than the effect of grafting the polymer to nanoparticles as a spherical brush.

## 4. Conclusions

A set of well-defined SPION with spherical PiPOx brushes was prepared using state-of-the-art techniques. By keeping the core diameter and the grafting density constant, the influence of the PiPOx molecular weight on the thermoresponsiveness of the core-shell nanoparticle dispersions could be studied in detail by DLS and DSC. All of the particles displayed reversible aggregation/deaggregation in water. Partial rehydration of the shell, investigated by DSC, proceeded on the experimental (minute) time scale, while spontaneous colloidal redispersion, investigated by DLS, was generally reversible only on a much longer (hour) time scale. For both free polymers and spherical brushes, the CSTs decreased and transition enthalpies increased with molecular weight. A reduction in the CST and transition enthalpy was observed after grafting to SPION, confirming the strong impact of polymer topology on the thermoresponsive properties. The reduction in CST observed for PiPOx-grafted SPION was more pronounced than for analogous systems based on PNIPAM. The PiPOx-grafted brushes displayed broadened transitions in DSC, which could be attributed to a main transition at a lower temperature for the part of the brush closest to the core that is identical for all molecular weights and a second distinct transition at a higher temperature, observed only for high molecular weight grafted PiPOx corresponding to a mushroom-like state in the outer shell. Importantly, the thermally-induced aggregation of thermoresponsive SPION in terms of cluster size appeared to be controlled by the outer shell transition.

The addition of kosmotropic salts resulted in a reduction of CSTs for PiPOx-grafted SPION with anions having a stronger impact than cations. Aggregation in pure water led to the formation of small clusters consisting of only a few SPION. By contrast, the addition of kosmotropes enabled sedimentation of PiPOx-grafted SPION upon heating, which would facilitate easy magnetic removal of the material, a crucial feature for potential applications in the areas of catalysis, cell separation, and molecular fishing. Consequently, designing thermoresponsive core-shell nanoparticles for a specific application requires taking the dispersion medium into account, as the CSTs of free polymers and spherical brushes in water are misguiding. Poly(2-oxazolines) possess the advantage that the highlighted effects can be compensated by the choice of side chains and monomer composition of the grafted polymer to design both the CST and tune colloidal stability as desired for applications.

## Figures and Tables

**Table 1:** Characteristics of poly(2-isopropyl-2-oxazoline) (PiPOx)-grafted superparamagnetic iron oxide nanoparticles.

Sample	Core-Diameter [nm]	Molecular Weight (GPC) [kg mol^−1^]		TGA		DLS $D_{H}$ [nm] *
			Weight Loss [wt %]	Residue [wt %]	*σ* [Molecules nm^−^²]	
FeOx-6	9.1 ± 0.3	6	56	44	1.0	16.0 ± 2.9
FeOx-14	9.1 ± 0.3	14	70	30	0.8	17.2 ± 2.2
FeOx-21	9.1 ± 0.3	21	74	26	1.0	18.6 ± 2.3
FeOx-33	9.1 ± 0.3	33	87	13	1.0	20.2 ± 2.4

* In Milli-Q, averaging values in the hydrated shell state below the transition.

**Table 2:** Characteristics of free linear PiPOx coils.

Sample	Molecular Weight(GPC) [kg mol^−1^]	PDI	CST (DLS) [°C] *	CST (DSC) [°C] *		$\Delta H_{monomer\;}$* [kJ mol^−1^]
				Onset	Maximum	
PiPOx-6	6	1.08	46	51	65	2.1
PiPOx-14	14	1.05	43	43	49	4.2
PiPOx-21	21	1.10	39	40	43	4.3
PiPOx-33	33	1.15	37	38	39	4.2

* CST and transition enthalpy were measured in pure water.

**Table 3:** Hydrodynamic diameter ($D_{H}$) and CST of SPION grafted with PiPOx of different molecular weight at a concentration of 1 g L^−1^ in Milli-Q measured by DLS.

Sample	$D_{H,T<CST\;}$ [nm]	$D_{H,T>CST\;}$ [nm]	CST [°C]
FeOx-6	16.0 ± 2.9	19.9 ± 2.3	40
FeOx-14	17.2 ± 2.2	20.7 ± 1.6	38
FeOx-21	18.6 ± 2.3	31.8 ± 2.6	36
FeOx-33	20.2 ± 2.4	29.5 ± 4.7 *	35

* Values between 39 and 44 °C were considered for the calculation. It represented the metastable phase between two transitions.

**Table 4:** Hydrodynamic diameter ($D_{H}$) and CST of SPION grafted with PiPOx of different molecular weight at a concentration of 5 × 10^13^ particles per mL in Milli-Q water measured by DLS.

Sample	Concentration [g L^−1^]	$D_{H,T<CST\;}$ [nm]	$D_{H,T>CST\;}$ [nm]	CST [°C]
**FeOx-6**	0.23	15.8 ± 2.9	17.8 ± 3.2	44
**FeOx-14**	0.34	18.9 ± 1.6	97 ± 44	42
**FeOx-21**	0.54	19.0 ± 2.1	26.3 ± 3.4	37
**FeOx-33**	0.78	18.8 ± 3.5	27.2 ± 5.6 *	36

* Values between 35 and 40 °C were considered for the calculation. It represented the metastable phase between two transitions.

**Table 5:** DSC results for SPION grafted with PiPOx of different molecular weight.

Sample	CST [°C]		$\Delta H_{tot}$ [kJ mol^−1^]	$\Delta H_{monomer}$ * [kJ mol^−^^1^]	Degree of Polymerisation
	Onset	Maximum			
FeOx-6	43	49	19	0.4	51
FeOx-14	31	45	93	0.8	119
FeOx-21	30	40	448	2.5	181
FeOx-33	31	39	639	2.2	288

* Transition enthalpies, Δ were calculated by integration of the endothermic peak. $\Delta H_{monomer}$ is the normalized value of the transition enthalpy per monomer unit.

**Table 6:** Comparison of the DSC results for free linear PiPOx and SPION grafted with PiPOx of different molecular weight, measured for heating and cooling sweeps.

Sample	$T_{max,\;heating}$ [°C]	$T_{max,\;cooling}$ [°C]	$\Delta T_{max}$ [°C]	$\Delta H_{tot,\;heating}$ [kJ/mol]	$\Delta H_{tot,\;cooling}$ [kJ/mol]	$\frac{\Delta H_{tot,\;cooling}}{\Delta H_{tot,\;heating}}$ [%]
PiPOx-6	65	56	9	111	95	86
PiPOx-14	49	45	4	498	579	116
PiPOx-21	43	41	2	776	948	122
PiPOx-33	39	39	0	1220	1150	94
FeOx-6	49	45	4	19	4	21
FeOx-14	45	37	8	93	44	47
FeOx-21	40	40	0	448	373	83
FeOx-33	39	39	0	639	250	39

## Supplementary Materials

The following are available online at http://www.mdpi.com/2073-4360/10/4/451/s1, Figure S1: FTIR spectra of SPION samples, Figure S2: Transmission electron micrographs of SPION grafted with PiPOx of different MW, Figure S3: TGA curves of all SPION samples, Figure S4: Temperature cycled DLS of PiPOx grafted SPION measured at a concentration of 5 × 10^13^ particles per mL, Figure S5: DSC curves of free polymer PiPOx samples, Figure S6: DSC curves of iron oxide core shell nanoparticles, Figure S7: Colloidal stability of FeOx-21 at a concentration of 1 g L^−1^ in Milli-Q water and NaCl solution (0.1 M), Figure S8: Temperature-cycled DLS for FeOx-33 dispersions in Milli-Q at a concentration of 1g L^−1^: hydrodynamic diameter ($D_{H}$) vs. temperature of the heating curve is enlarged, Figure S9: DLS-heating curves for SPION dispersions (FeOx-21, 1 g L^−1^) with different concentrations of CaCl_2_, Figure S10: DLS-heating curves for SPION dispersions (FeOx-21, 1 g L^−1^) with different concentrations of KCl, Figure S11: DLS-heating curves for SPION dispersions (FeOx-21, 1 g L^−1^) with different concentrations of NaH_2_PO_4_, Figure S12: DLS-heating curves for SPION dispersions (FeOx-21, 1 g L^−1^) with different concentrations of MgCl_2_, Figure S13: DLS-heating curves for SPION dispersions (FeOx-21, 1 g L^−1^) with different concentrations of NaHCO_3_.

## References

[1] Kakwere H., Leal M.P., Materia M.E., Curcio A., Guardia P., Niculaes D., Marotta R., Falqui A., Pellegrino T. (2015). Functionalization of strongly interacting magnetic nanocubes with (thermo)responsive coating and their application in hyperthermia and heat-triggered drug delivery. ACS Appl. Mater. Interfaces.

[2] Liu C., Guo J., Yang W., Hu J., Wang C., Fu S. (2009). Magnetic mesoporous silica microspheres with thermo-sensitive polymer shell for controlled drug release. J. Mater. Chem..

[3] Zhang J., Misra R.D.K. (2007). Magnetic drug-targeting carrier encapsulated with thermosensitive smart polymer: Core-shell nanoparticle carrier and drug release response. Acta Biomater..

[4] Yildiz I., Sizirici Yildiz B. (2015). Applications of Thermoresponsive Magnetic Nanoparticles. J. Nanomater..

[5] Wang C., Irudayaraj J. (2010). Multifunctional magnetic-optical nanoparticle probes for simultaneous detection, separation, and thermal ablation of multiple pathogens. Small.

[6] Zhao Q., Chen N., Zhao D., Lu X. (2013). Thermoresponsive magnetic nanoparticles for seawater desalination. ACS Appl. Mater. Interfaces.

[7] Crassous J.J., Mihut A.M., Dietsch H., Pravaz O., Ackermann-Hirschi L., Hirt A.M., Schurtenberger P. (2014). Advanced multiresponsive comploids: From design to possible applications. Nanoscale.

[8] Luxenhofer R., Sahay G., Schulz A., Alakhova D., Bronich T.K., Jordan R., Kabanov A.V. (2011). Structure-property relationship in cytotoxicity and cell uptake of poly(2-oxazoline) amphiphiles. J. Control. Release.

[9] Schellekens H., Hennink W.E., Brinks V. (2013). The immunogenicity of polyethylene glycol: Facts and fiction. Pharm. Res..

[10] Luxenhofer R., Han Y., Schulz A., Tong J., He Z., Kabanov A.V., Jordan R. (2012). Poly(2-oxazoline)s as Polymer Therapeutics. Macromol. Rapid Commun..

[11] Glassner M., Lava K., De La Rosa V.R., Hoogenboom R. (2014). Tuning the LCST of poly(2-cyclopropyl-2-oxazoline) via gradient copolymerization with 2-ethyl-2-oxazoline. J. Polym. Sci. Part A Polym. Chem..

[12] Park J.S., Kataoka K. (2006). Precise control of lower critical solution temperature of thermosensitive poly(2-isopropyl-2-oxazoline) via gradient copolymerization with 2-ethyl-2-oxazoline as a hydrophilic comonomer. Macromolecules.

[13] Hoogenboom R., Thijs H.M.L., Jochems M.J.H.C., van Lankvelt B.M., Fijten M.W.M., Schubert U.S. (2008). Tuning the LCST of poly(2-oxazoline)s by varying composition and molecular weight: Alternatives to poly(*N*-isopropylacrylamide)?. Chem. Commun..

[14] Verbraeken B., Monnery B.D., Lava K., Hoogenboom R. (2017). The chemistry of poly(2-oxazoline)s. Eur. Polym. J..

[15] Uyama H., Kobayashi S. (1992). A Novel Thermo-Sensitive Polymer. Poly(2-iso-propyl-2-oxazoline). Chem. Lett..

[16] Hoogenboom R., Schlaad H. (2017). Thermoresponsive poly(2-oxazoline)s, polypeptoids, and polypeptides. Polym. Chem..

[17] Zhao J., Hoogenboom R., Van Assche G., Van Mele B. (2010). Demixing and remixing kinetics of poly(2-isopropyl-2-oxazoline) (PIPOZ) aqueous solutions studied by modulated temperature differential scanning calorimetry. Macromolecules.

[18] Aseyev V., Tenhu H., Winnik F.M. (2010). Non-ionic Thermoresponsive Polymers in Water. Romanian Reports of Physics.

[19] Park J.S., Akiyama Y., Winnik F.M., Kataoka K. (2004). Versatile synthesis of end-functionalized thermosensitive poly(2-isopropyl-2-oxazolines). Macromolecules.

[20] Salzinger S., Huber S., Jaksch S., Busch P., Jordan R., Papadakis C.M. (2012). Aggregation behavior of thermo-responsive poly(2-oxazoline)s at the cloud point investigated by FCS and SANS. Colloid Polym. Sci..

[21] Bloksma M.M., Bakker D.J., Weber C., Hoogenboom R., Schubert U.S. (2010). The effect of hofmeister salts on the LCST transition of poly(2-oxazoline)s with varying hydrophilicity. Macromol. Rapid Commun..

[22] Huber S., Hutter N., Jordan R. (2008). Effect of end group polarity upon the lower critical solution temperature of poly(2-isopropyl-2-oxazoline). Colloid Polym. Sci..

[23] Diab C., Akiyama Y., Kataoka K., Winnik F.M. (2004). Microcalorimetric Study of the Temperature-Induced Phase Separation in Aqueous Solutions of Poly(2-isopropyl-2-oxazolines). Macromolecules.

[24] Huber S., Jordan R. (2008). Modulation of the lower critical solution temperature of 2-Alkyl-2-oxazoline copolymers. Colloid Polym. Sci..

[25] Tatar Güner P., Demirel A.L. (2012). Effect of Anions on the Cloud Point Temperature of Aqueous Poly(2-ethyl-2-oxazoline) Solutions. J. Phys. Chem. B.

[26] Amirova A.I., Dudkina M.M., Tenkovtsev A.V., Filippov A.P. (2014). Self-assembly of star-shaped poly(2-isopropyl-2-oxazoline) in aqueous solutions. Colloid Polym. Sci..

[27] Zhang N., Luxenhofer R., Jordan R. (2012). Thermoresponsive Poly(2-Oxazoline) Molecular Brushes by Living Ionic Polymerization: Modulation of the Cloud Point by Random and Block Copolymer Pendant Chains. Macromol. Chem. Phys..

[28] Zhang N., Huber S., Schulz A., Luxenhofer R., Jordan R. (2009). Cylindrical Molecular Brushes of Poly(2-oxazoline)s from 2-Isopropenyl-2-oxazoline. Macromolecules.

[29] Zhang N., Luxenhofer R., Jordan R. (2012). Thermoresponsive Poly(2-oxazoline) Molecular Brushes by Living Ionic Polymerization: Kinetic Investigations of Pendant Chain Grafting and Cloud Point Modulation by Backbone and Side Chain Length Variation. Macromol. Chem. Phys..

[30] Grünewald T.A., Lassenberger A., van Oostrum P.D.J., Rennhofer H., Zirbs R., Capone B., Vonderhaid I., Amenitsch H., Lichtenegger H.C., Reimhult E. (2015). Core–Shell Structure of Monodisperse Poly(ethylene glycol)-Grafted Iron Oxide Nanoparticles Studied by Small-Angle X-ray Scattering. Chem. Mater..

[31] Kurzhals S., Gal N., Zirbs R., Reimhult E. (2017). Controlled aggregation and cell uptake of thermoresponsive polyoxazoline-grafted superparamagnetic iron oxide nanoparticles. Nanoscale.

[32] Hyeon T., Lee S.S., Park J., Chung Y., Na H.B. (2001). Synthesis of highly crystalline and monodisperse maghemite nanocrystallites without a size-selection process. J. Am. Chem. Soc..

[33] Zirbs R., Lassenberger A., Vonderhaid I., Kurzhals S., Reimhult E. (2015). Melt-grafting for the synthesis of core–shell nanoparticles with ultra-high dispersant density. Nanoscale.

[34] Witte H., Seeliger W. (1974). Cyclische Imidsäureester aus Nitrilen und Aminoalkoholen. Justus Liebigs Ann. Chem..

[35] Monnery B.D., Shaunak S., Thanou M., Steinke J.H.G. (2015). Improved synthesis of linear poly(ethylenimine) via low-temperature polymerization of 2-isopropyl-2-oxazoline in chlorobenzene. Macromolecules.

[36] Mondini S., Ferretti A.M., Puglisi A., Ponti A. (2012). Pebbles and PebbleJuggler: Software for accurate, unbiased, and fast measurement and analysis of nanoparticle morphology from transmission electron microscopy (TEM) micrographs. Nanoscale.

[37] Amstad E., Gillich T., Bilecka I., Textor M., Reimhult E. (2009). Ultrastable Iron Oxide Nanoparticle Colloidal Suspensions Using Dispersants with Catechol-Derived Anchor Groups. Nano Lett..

[38] Yuen A.K.L., Hutton G.A., Masters A.F., Maschmeyer T. (2012). The interplay of catechol ligands with nanoparticulate iron oxides. Dalton Trans..

[39] Wang B., Wei Q., Qu S. (2013). Synthesis and characterization of uniform and crystalline magnetite nanoparticles via oxidation-precipitation and modified co-precipitation methods. Int. J. Electrochem. Sci..

[40] Amstad E., Gehring A.U., Fischer H., Nagaiyanallur V.V., Hähner G., Textor M., Reimhult E. (2011). Influence of Electronegative Substituents on the Binding Affinity of Catechol-Derived Anchors to Fe_3_O_4_ Nanoparticles. J. Phys. Chem. C.

[41] Katsumoto Y., Tsuchiizu A., Qiu X., Winnik F.M. (2012). Dissecting the mechanism of the heat-induced phase separation and crystallization of poly(2-isopropyl-2-oxazoline) in water through vibrational spectroscopy and molecular orbital calculations. Macromolecules.

[42] Furuncuoğlu Özaltın T., Aviyente V., Atılgan C., Demirel L. (2017). Multiscale modeling of poly(2-isopropyl-2-oxazoline) chains in aqueous solution. Eur. Polym. J..

[43] Li T., Tang H., Wu P. (2015). Molecular Evolution of Poly(2-isopropyl-2-oxazoline) Aqueous Solution during the Liquid–Liquid Phase Separation and Phase Transition Process. Langmuir.

[44] Sun S., Wu P. (2015). From globule to crystal: A spectral study of poly(2-isopropyl-2-oxazoline) crystallization in hot water. Phys. Chem. Chem. Phys..

[45] Kurzhals S., Gal N., Zirbs R., Reimhult E. (2017). Aggregation of thermoresponsive core-shell nanoparticles: Influence of particle concentration, dispersant molecular weight and grafting. J. Colloid Interface Sci..

[46] Vasicek T.W., Jenkins S.V., Vaz L., Chen J., Stenken J.A. (2017). Thermoresponsive nanoparticle agglomeration/aggregation in salt solutions: Dependence on graft density. J. Colloid Interface Sci..

[47] Koshkina O., Lang T., Thiermann R., Docter D., Stauber R.H., Secker C., Schlaad H., Weidner S., Mohr B., Maskos M. (2015). Temperature-Triggered Protein Adsorption on Polymer-Coated Nanoparticles in Serum. Langmuir.

[48] Chen N., Xiang X., Heiden P.A. (2013). Tuning thermoresponsive behavior of diblock copolymers and their gold core hybrids. Part 2. How properties change depending on block attachment to gold nanoparticles. J. Colloid Interface Sci..

[49] Kurzhals S., Pretzner B., Reimhult E., Zirbs R. (2017). Thermoresponsive Polypeptoid-Coated Superparamagnetic Iron Oxide Nanoparticles by Surface-Initiated Polymerization. Macromol. Chem. Phys..

[50] Xu J., Liu S. (2009). Synthesis of well-defined 7-arm and 21-arm poly(*N*-isopropylacrylamide) star polymers with β-cyclodextrin cores via click chemistry and their thermal phase transition behavior in aqueous solution. J. Polym. Sci. Part A Polym. Chem..

[51] Kurzhals S., Schroffenegger M., Gal N., Zirbs R., Reimhult E. (2017). Influence of Grafted Block Copolymer Structure on Thermoresponsiveness of Superparamagnetic Core–Shell Nanoparticles. Biomacromolecules.

[52] Lo Verso F., Egorov S.A., Milchev A., Binder K. (2010). Spherical polymer brushes under good solvent conditions: Molecular dynamics results compared to density functional theory. J. Chem. Phys..

[53] Shan J., Chen J., Nuopponen M., Tenhu H. (2004). Two Phase Transitions of Poly(*N*-isopropylacrylamide) Brushes Bound to Gold Nanoparticles. Langmuir.

