# Supplementary material for: The Role of Chain Molecular Weight and Hofmeister Series Ions in Thermal Aggregation of Poly(2-Isopropyl-2-Oxazoline) Grafted Nanoparticles
Source: Polymers (Basel). 2018 Apr 17;10(4):451. doi: 10.3390/polym10040451 (PMC6415256; doi:10.3390/polym10040451)
Supplement: Supplementary file 1 [file polymers-10-00451-s001.pdf]

polymers

MDPI

*Supporting Information*

# The role of chain molecular weight and Hofmeister series ions in thermal aggregation of poly(2-isopropyl-2-oxazoline) grafted nanoparticles

**Martina Schroffenegger[1], Ronald Zirbs[1], Steffen Kurzhals[1] and Erik Reimhult[1,*]**

[1] University of Natural Resources and Life Sciences Vienna, Muthgasse 11, 1190 Vienna, Austria; martina.schroffenegger@boku.ac.at (M.S.); ronald.zirbs@boku.ac.at (R.Z.); steffen.kurzhals@boku.ac.at (S.K.)
* Correspondence: erik.reimhult@boku.ac.at; Tel.: +43 1 47654-80211

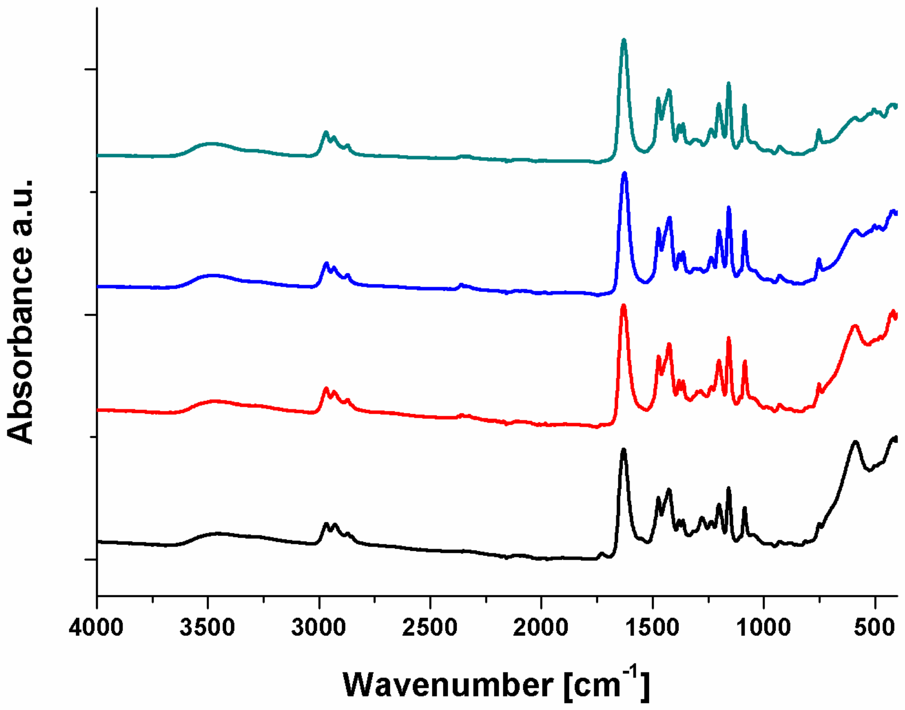


**Figure S1.** FTIR spectra of SPION samples. Black: FeOx-6, red: FeOx-14, blue: FeOx-21 and cyan: FeOx-33.

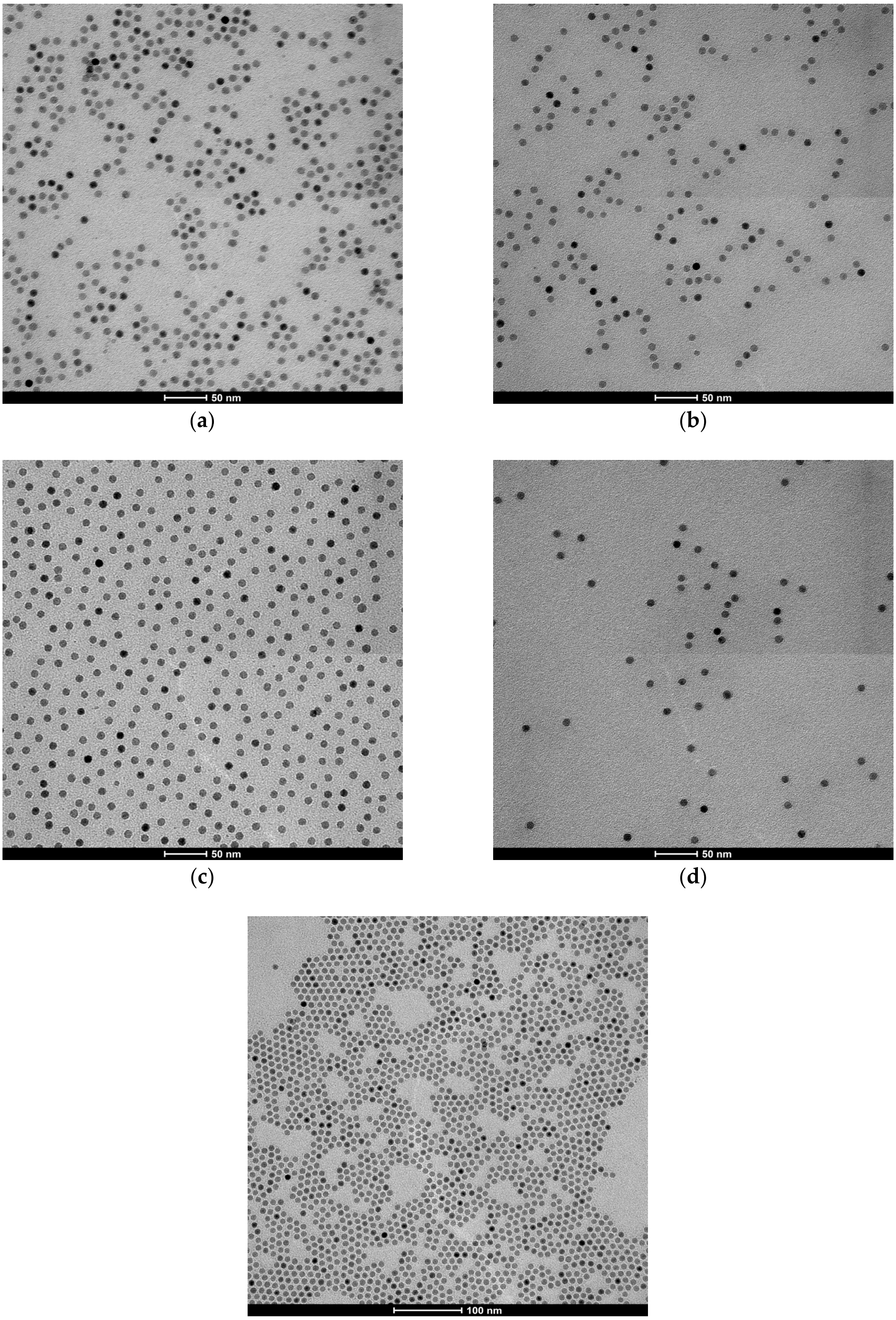


(**a**) (**b**) (**c**) (**d**) (**e**)

**Figure S2.** Transmission electron micrographs of 9.1 nm diameter SPION grafted with PiPOx of different MW, (**a**): FeOx-6 (grafted with PiPOx 6 kg mol$^{-1}$), (**b**): FeOx-14 (grafted with PiPOx 14 kg mol$^{-1}$), (**c**): FeOx-21 (grafted with PiPOx 21 kg mol$^{-1}$) and (**d**): FeOx-33 (grafted with PiPOx 33 kg mol$^{-1}$), (**e**) oleic acid coated SPION.

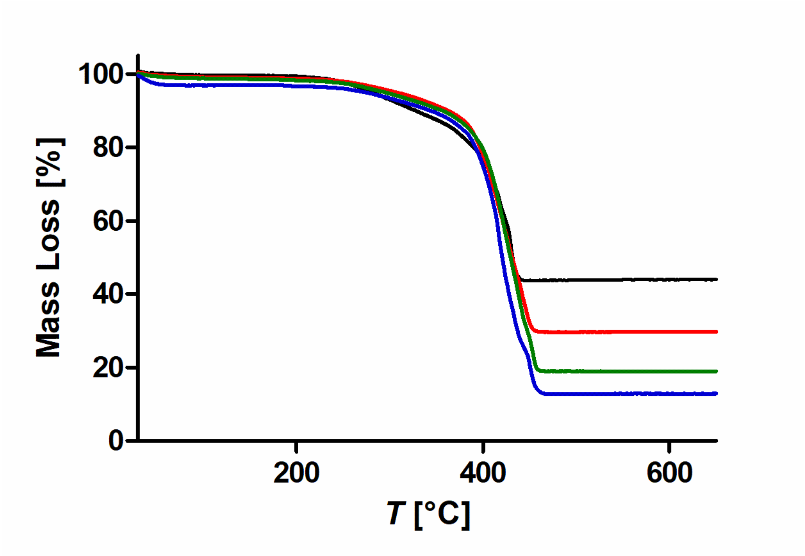


**Figure S3.** TGA curves of all SPION samples. TGA was measured with a heating rate of 10 °C $min^{-1}$ at a constant flow of 80 mL $min^{-1}$ of synthetic air. Black: FeOx-6, red: FeOx-14, green: FeOx-21, blue: FeOx-33.

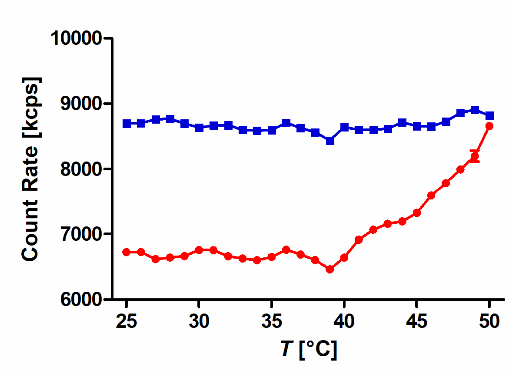


(**a**)

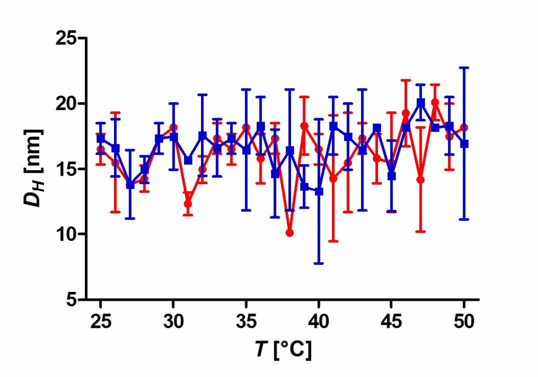


(**b**)

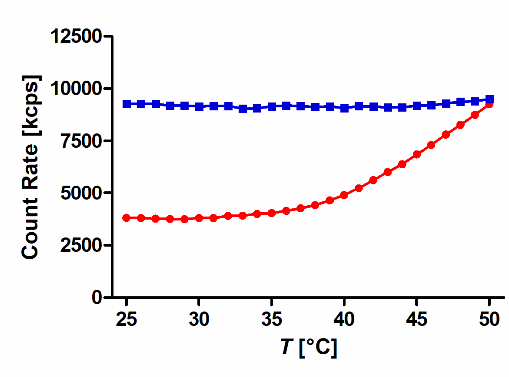


(**c**)

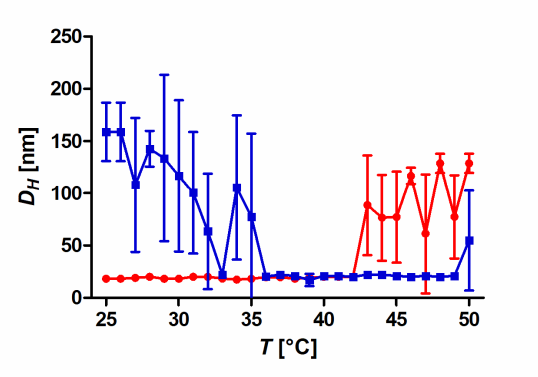


(**d**)

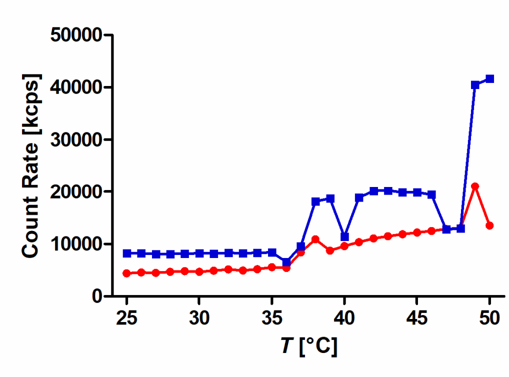


(**e**)

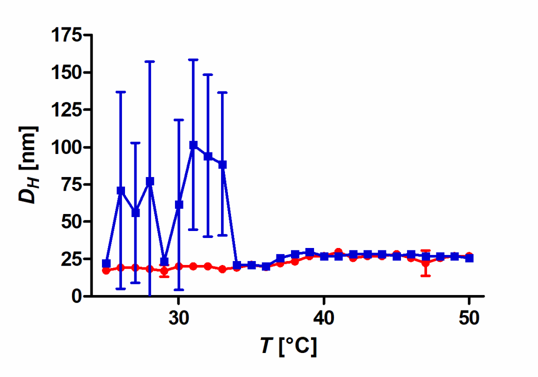


(**f**)

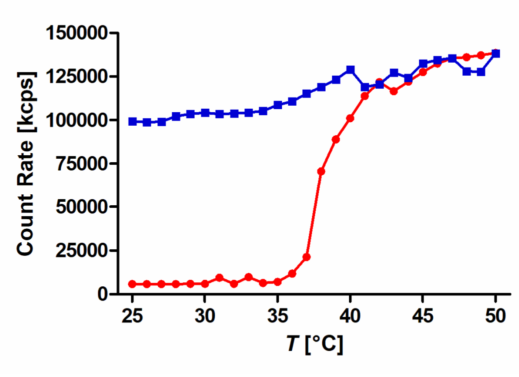


(**g**)

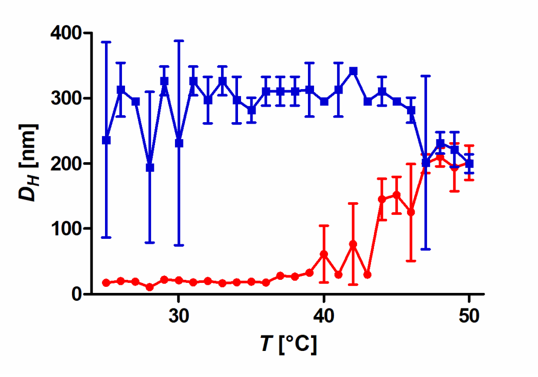


(**h**)

**Figure S4.** Temperature-cycled DLS of PiPOx grafted SPION dispersions measured at a concentration of $5{\cdot}10^{13}$ particles per mL: FeOx-6: (**a**) and (**b**), FeOx-14: (**c**) and (**d**), FeOx-21: (**e**) and (**f**), FeOx-33: (**g**) and (**h**). Left: count rate *vs* temperature, right: hydrodynamic diameter ($D_H$) *vs* temperature. In red circles: heating curve, in blue squares: cooling curve. Mean values and standard deviation of count rate and number weighted diameter were calculated from three measurements for each temperature step.

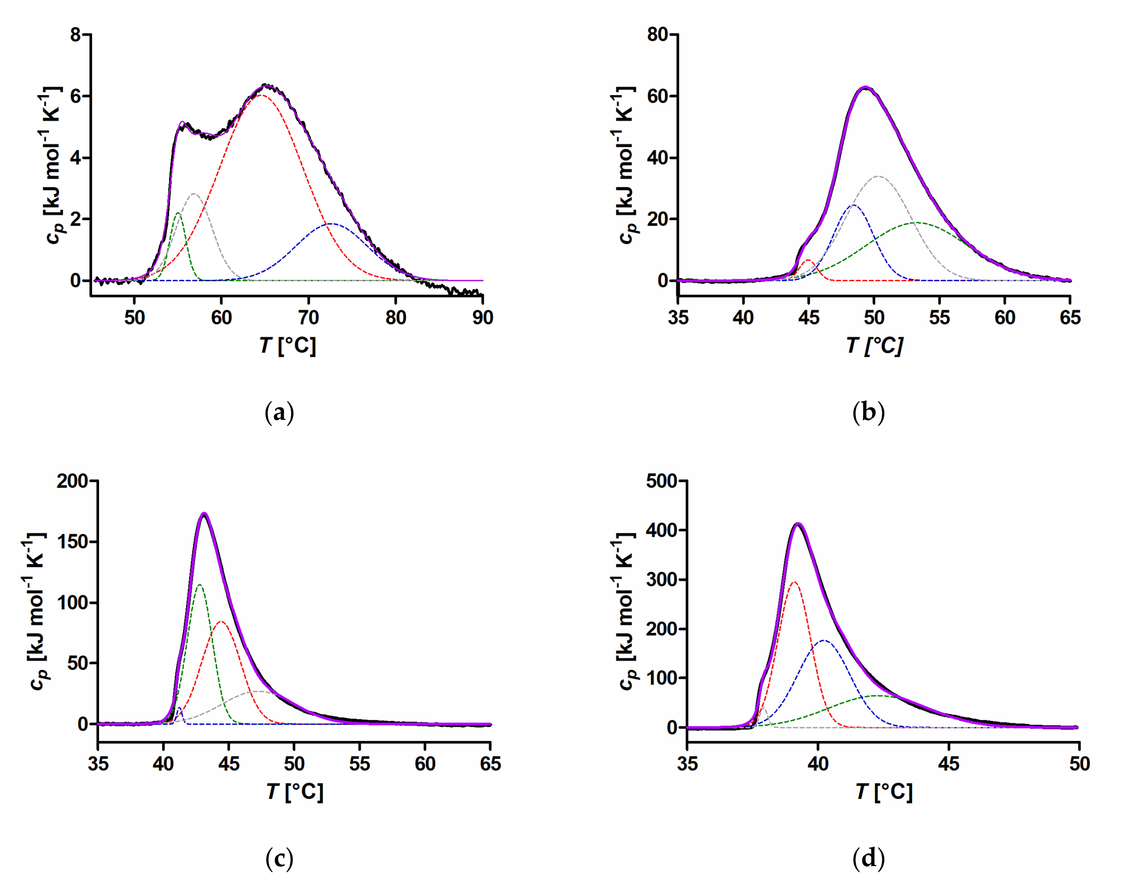


**Figure S5.** DSC curves of free polymer PiPOx samples. The samples were measured with a concentration of 1 g L$^{-1}$ in Milli-Q water with a heating rate of 60 °C h$^{-1}$. (**a**): PiPOx-6, (**b**): PiPOx-14, (**c**): PiPOx-21 and (**d**): PiPOx-33. Black: raw data of the measurements, dashed lines: fitted curves, violet: sum of fitted curves.

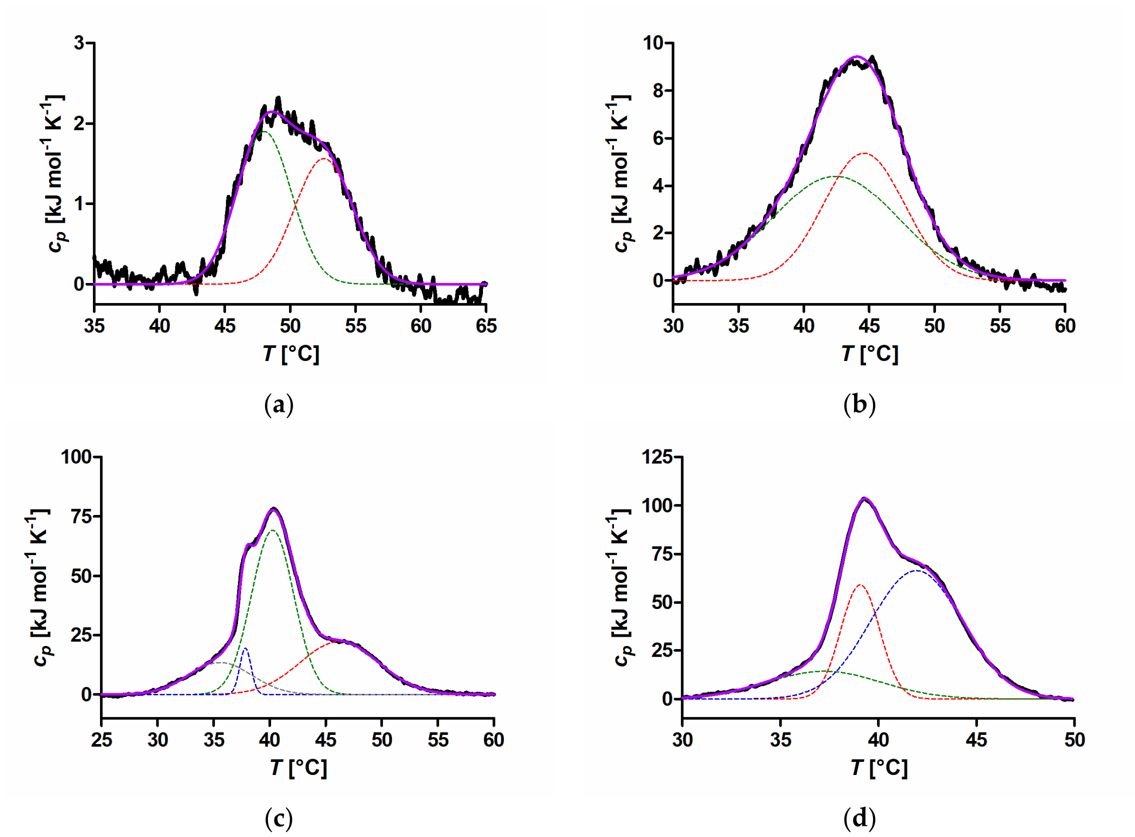


**Figure S6.** DSC curves of core-shell iron oxide nanoparticles. The samples were measured with a concentration of 1 g L$^{-1}$ in Milli-Q water with a heating rate of 60 °C h$^{-1}$. (**a**): FeOx-6, (**b**): FeOx-14, (**c**): FeOx-21 and (**d**): FeOx-33. Black: raw data of the measurements, dashed lines: fitted curves, violet: sum of fitted curves.

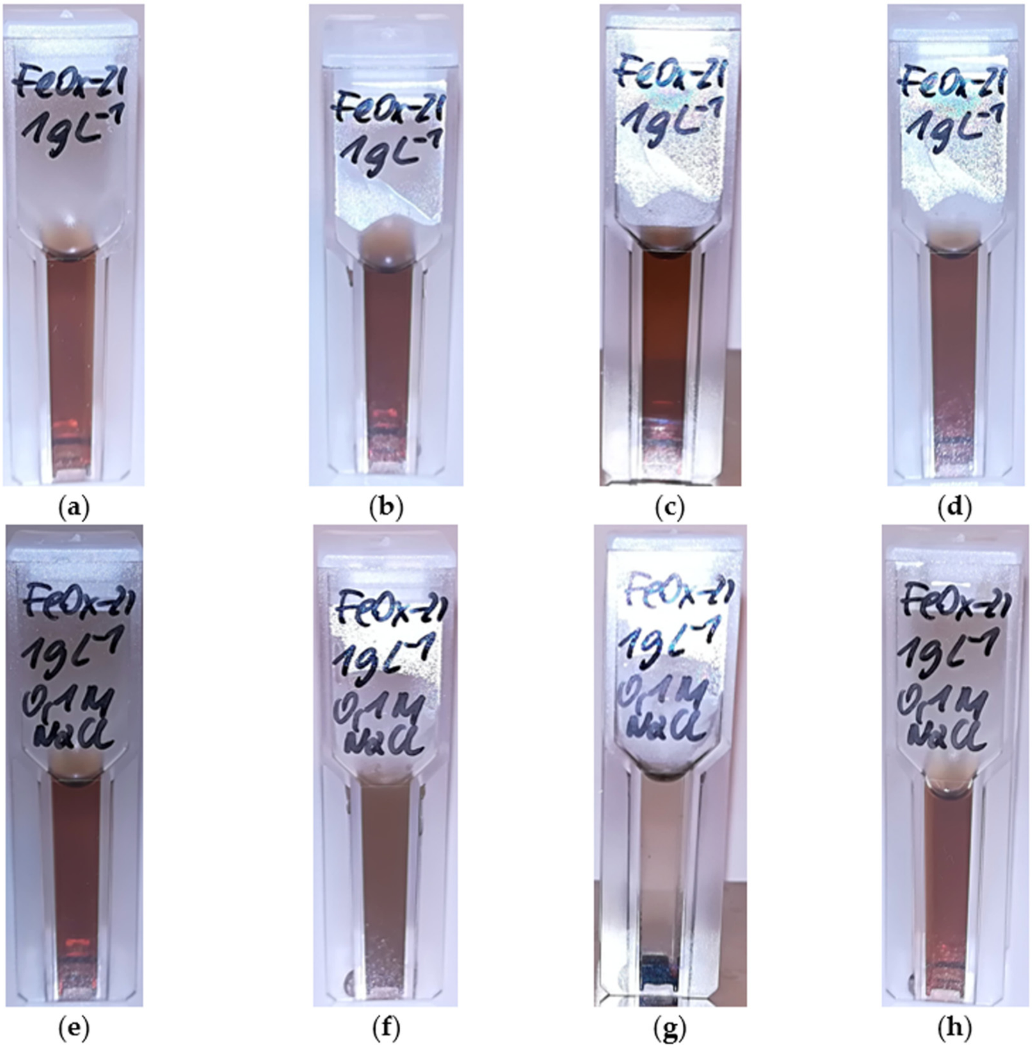


**Figure S7.** Photographs of colloidal stability of core-shell SPION FeOx-21 dispersions at a concentration of 1 g $L^{-1}$ in Milli-Q water. Upper row, without salt, lower row with a NaCl concentration of 0.1 M. (**a**) and (**e**) at room temperature, (**b**) and (**f**) at 50 °C, (**c**) and (**g**) at 50 °C on a static magnet (remanence = 1.29 T), (**d**) and (**h**) after cooling to room temperature.

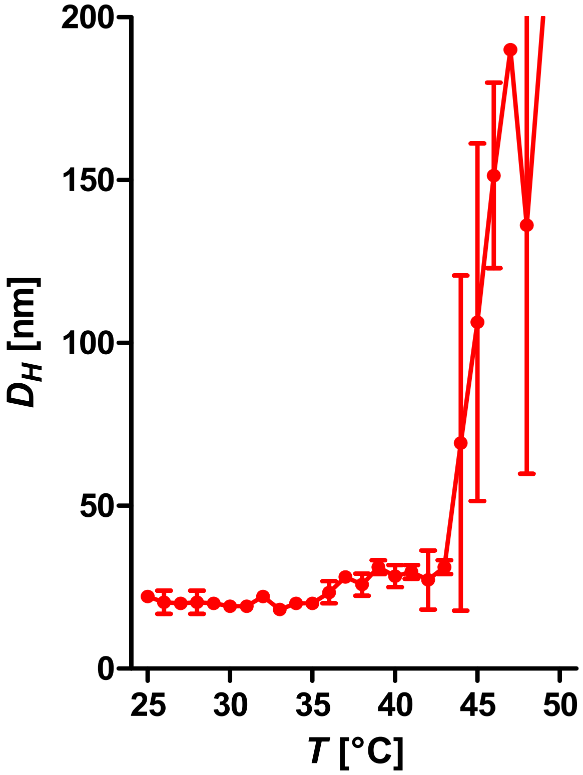


**Figure S8:** Temperature-cycled DLS for FeOx-33 dispersions in Milli-Q at a concentration of 1g $L^{-1}$: hydrodynamic diameter ($D_H$) *vs* temperature of the heating curve is enlarged.

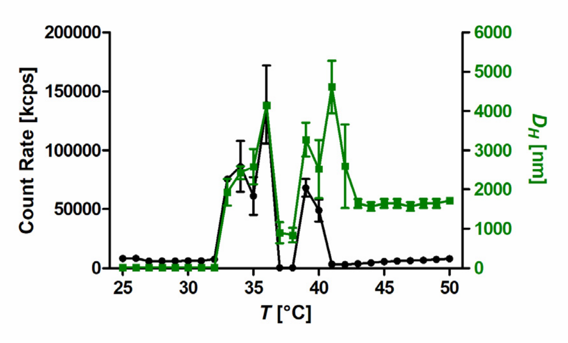


(**a**)

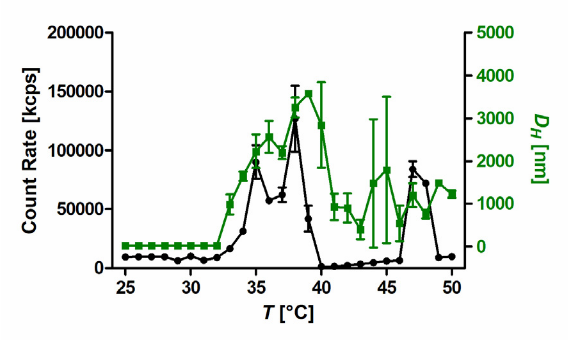


(**b**)

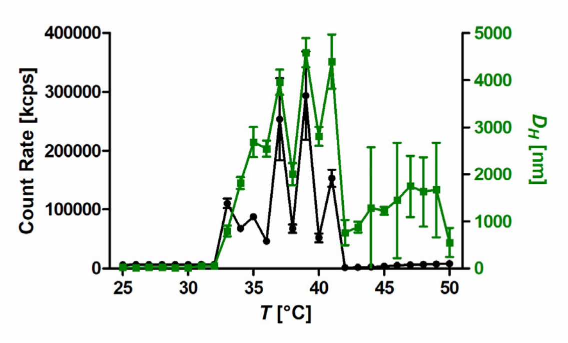


(**c**)

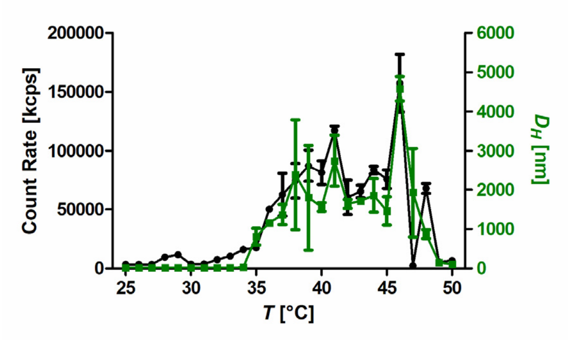


(**d**)

**Figure S9.** DLS-heating curves for core-shell SPION (FeOx-21, 1g $L^{-1}$) dispersions with different concentrations of $CaCl_2$. (**a**): 0.01 M $CaCl_2$, (**b**): 0.05 M $CaCl_2$, (**c**): 0.1 M $CaCl_2$, (**d**): 0.16 M $CaCl_2$. Black: count rate and in green hydrodynamic diameter curve. Mean values and standard deviation of count rate and number weighted diameter were calculated from three measurements for each temperature step.

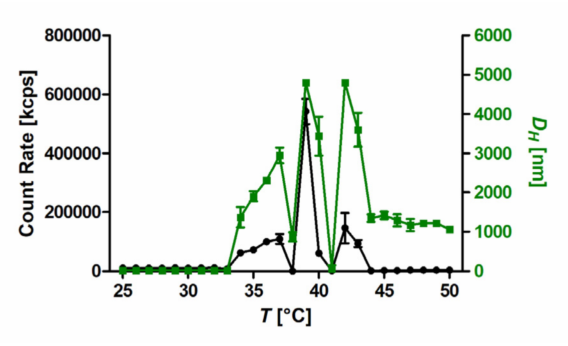


(**a**)

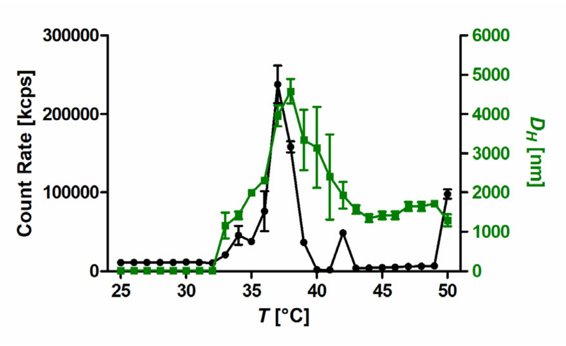


(**b**)

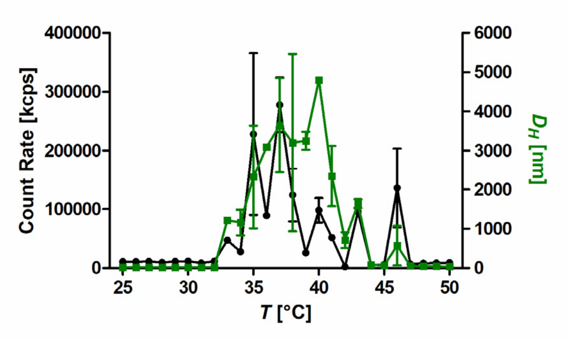


(**c**)

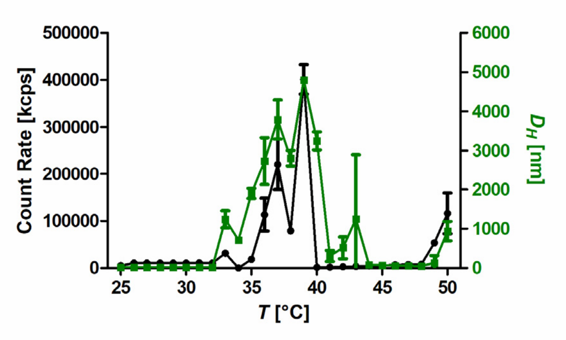


(**d**)

**Figure S10.** DLS-heating curves for core-shell SPION dispersions (FeOx-21, 1g $L^{-1}$) with different concentrations of KCl. (**a**): 0.01 M KCl, (**b**): 0.05 M KCl, (**c**): 0.1 M KCl, (**d**): 0.16 M KCl. Black: count rate and in green hydrodynamic diameter curve. Mean values and standard deviation of count rate and number weighted diameter were calculated from three measurements for each temperature step.

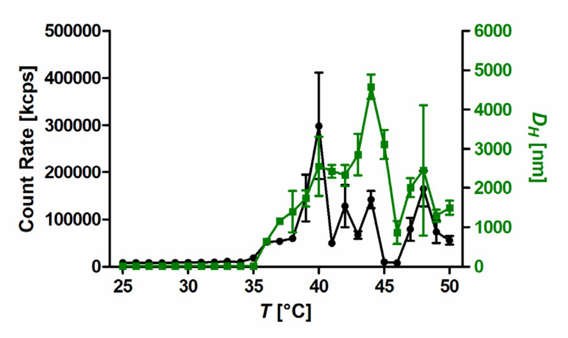


(**a**)

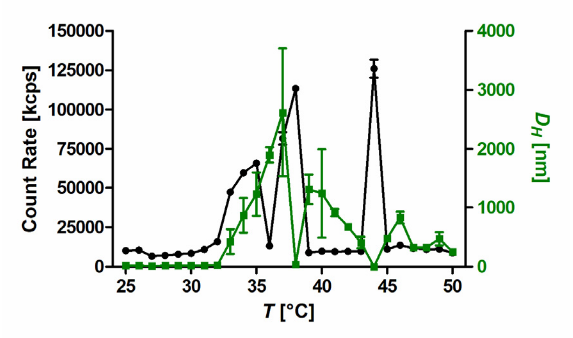


(**b**)

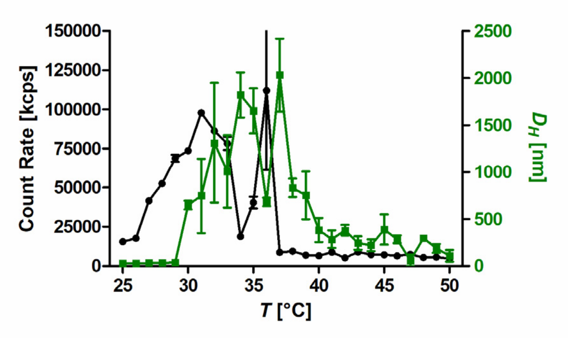


(**c**)

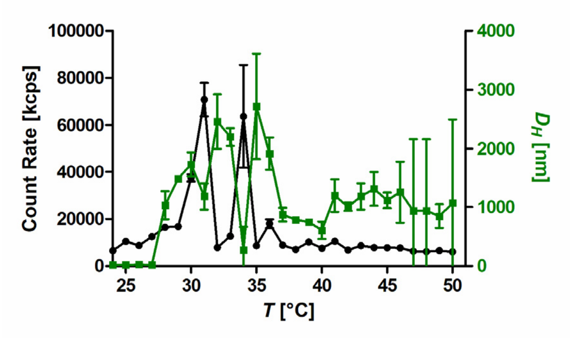


(**d**)

**Figure S11.** DLS-heating curves for core-shell SPION dispersions (FeOx-21, 1g $L^{-1}$) with different concentrations of $NaH_2PO_4$. (**a**): 0.01 M $NaH_2PO_4$, (**b**): 0.05 M $NaH_2PO_4$, (**c**): 0.1 M $NaH_2PO_4$, (**d**): 0.16 M $NaH_2PO_4$. Black: count rate and in green hydrodynamic diameter curve. Mean values and standard deviation of count rate and number weighted diameter were calculated from three measurements for each temperature step.

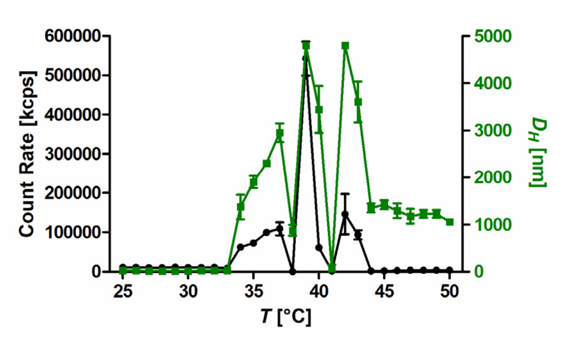


(**a**)

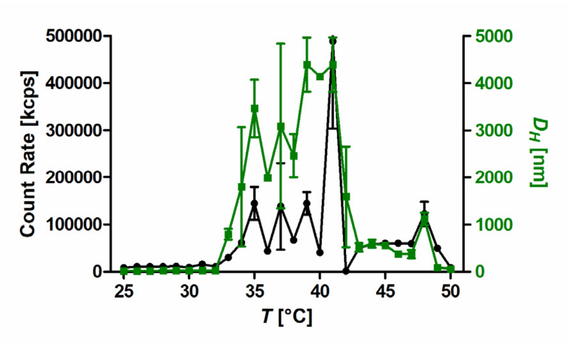


(**b**)

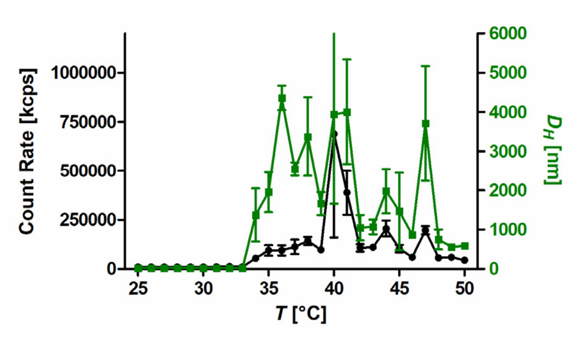


(**c**)

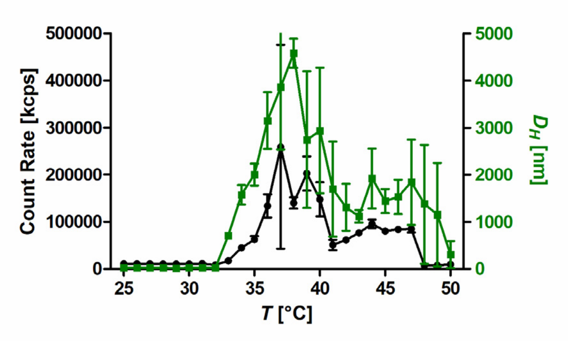


(**d**)

**Figure S12.** DLS-heating curves for core-shell SPION dispersions (FeOx-21, 1g $L^{-1}$) with different concentrations of $MgCl_2$. (**a**): 0.01 M $MgCl_2$, (**b**): 0.05 M $MgCl_2$, (**c**): 0.1 M $MgCl_2$, (**d**): 0.16 M $MgCl_2$. Black: count rate and in green hydrodynamic diameter curve. Mean values and standard deviation of count rate and number weighted diameter were calculated from three measurements for each temperature step.

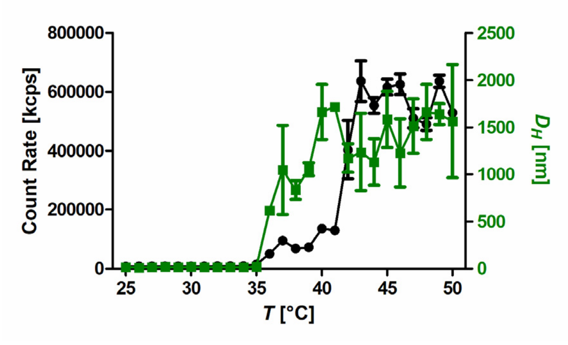


(**a**)

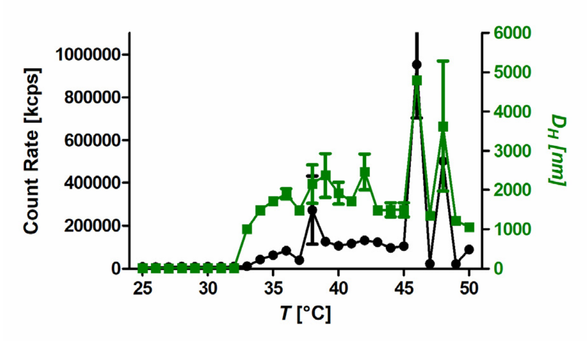


(**b**)

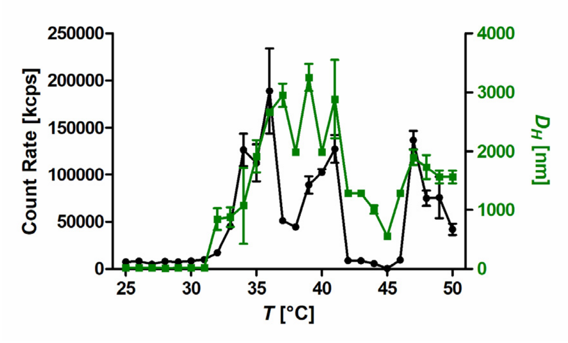


(**c**)

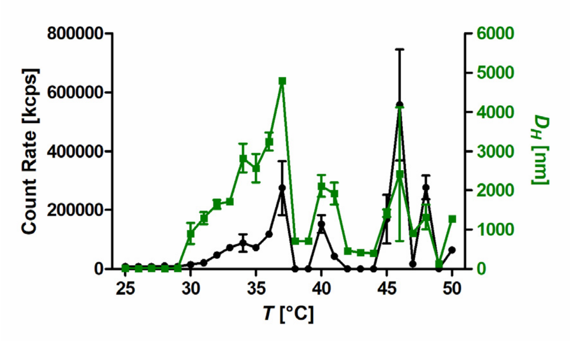


(**d**)

**Figure S13.** DLS-heating curves for core-shell SPION dispersions (FeOx-21, 1g $L^{-1}$) with different concentrations of $NaHCO_3$. (**a**): 0.01 M $NaHCO_3$, (**b**): 0.05 M $NaHCO_3$, (**c**): 0.1 M $NaHCO_3$, d: 0.16 M $NaHCO_3$. Black: count rate and in green hydrodynamic diameter curve. Mean values and standard deviation of count rate and number weighted diameter were calculated from three measurements for each temperature step.

© 2018 by the authors. Submitted for possible open access publication under the terms and conditions of the Creative Commons Attribution (CC BY) license (http://creativecommons.org/licenses/by/4.0/).
